# The kinase GSK-3 alters the RNA-binding protein landscape of lipid metabolism transcripts leading to altered expression in the *C. elegans* nervous system

**DOI:** 10.1093/nar/gkaf785

**Published:** 2025-08-19

**Authors:** Ananya Mahapatra, Meghana Mohankumar, Heather A Hundley

**Affiliations:** Genome, Cell and Developmental Biology Graduate Program, Indiana University, Bloomington, IN 47405, United States; Department of Chemistry, Indiana University, Bloomington, IN 47405, United States; Department of Biology, Indiana University, Bloomington, IN 47405, United States

## Abstract

Tissue-specific regulation of gene expression is essential for multicellular organisms, and RNA-binding proteins play central roles in these molecular processes. To determine how the *Caenorhabditis elegans* RNA-binding protein, ADR-1, regulates tissue-specific gene expression, we profiled the RNA-binding targets of ADR-1 in neural cells and assessed the effects of ADR-1 binding on neural gene expression. We identified a cohort of neural transcripts that function in lipid metabolism and are directly regulated by ADR-1 binding. To identify cellular factors that influence ADR-1 binding, a forward genetic screen was performed, revealing that the serine/threonine protein kinase, glycogen synthase kinase-3 (GSK-3), inhibits ADR-1 binding to the cohort. Further investigation revealed that the RNA-binding protein VIG-1 physically interacts with ADR-1, and the two proteins coordinately bind the neural lipid metabolism transcripts. Additional experiments revealed that VIG-1 is phosphorylated in a GSK-3-dependent manner, which inhibits the VIG-1–ADR-1 complex from binding the regulon in wild-type animals. Importantly, inhibition of GSK-3 kinase activity in wild-type animals also resulted in decreased neural expression of lipid metabolism genes. Together, we reveal that the interplay between a kinase and RNA-binding proteins regulates the expression of lipid metabolism genes within neural cells, potentially impacting stress resistance and longevity.

## Graphical Abstract

**Graphical Abstract:**
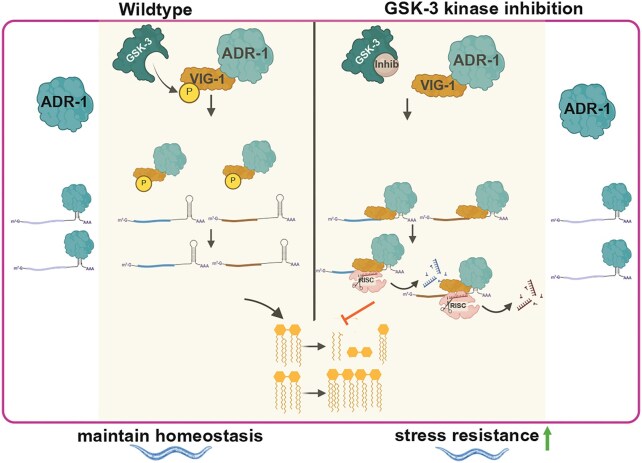

## Introduction

Post-transcriptional regulation is critical for proper gene expression in eukaryotic organisms and often goes awry in disease [1]. Central to post-transcriptional gene regulation are RNA-binding proteins (RBPs) [2]. RBPs can not only influence RNA metabolism individually but also interact with each other to form dynamic complexes that coordinate the processing and fate of RNA molecules [3].

While RBPs perform essential roles in all tissues, post-transcriptional gene regulation is particularly important in the nervous system due to the diversity of cell types, unique cellular architecture, and role of this tissue in sensing and responding to external stimuli [4]. To meet the demands of the nervous system, RBPs influence RNA metabolism through chemical modification of RNA, regulating splicing, impacting messenger RNA (mRNA) transport and stability as well as translational control [5]. Together, these processes generate the molecular diversity and spatial gene expression required for proper brain development and function [6]. Consistent with these important roles, RBP dysfunction occurs in a diverse range of neurological disorders and neurodegenerative diseases [7].

Adenosine deaminases that act on RNA (ADARs) are a class of double-stranded RNA (dsRNA) binding proteins that are essential for mammalian brain development [8, 9], with altered ADAR function observed in several neuropathological and neurodegenerative diseases including Alzheimer’s disease, Parkinson’s disease, epilepsy, and glioblastoma [10, 11]. The importance of ADAR function in the nervous system is evolutionarily conserved. In the fruit fly, *Drosophila*, proper ADAR activity is required for preventing age-dependent neurodegeneration, and flies lacking ADARs exhibit tremors and discoordination [12, 13]. In the nematode *Caenorhabditis elegans*, animals lacking ADARs display defects in sensing chemicals [14], which can be rescued by restoring altered expression of a neural gene [15].

ADAR function in the nervous system was first uncovered when single nucleotide differences between mammalian mRNAs and genomic information were observed for a number of neurotransmitter receptors and ion channels [16]. These alterations to the mRNA were a result of ADARs catalyzing the deamination of adenosine (A) to yield inosine (I), a process referred to as RNA editing [17]. A-to-I RNA editing occurs in the neural transcriptomes of all animals and contributes to both transcriptome and proteome diversity [18], with the pinnacle of editing observed in the nervous system of cephalopods where ∼100 000 A-to-I events in coding regions are thought to contribute to the extremely complex behavior observed in these invertebrates [19].

In addition to A-to-I editing, ADARs can also regulate gene expression via RNA binding [20]. The importance of editing-independent functions is underscored by the presence of ADAR homologs which lack the ability to catalyze adenosine deamination in organisms ranging from the sea slug to humans [21]. These deaminase-deficient proteins possess dsRNA-binding domains and can influence gene expression directly via binding mRNA.

In our prior work, we demonstrated that the editing-deficient *C. elegans* ADAR protein, ADR-1, specifically regulates expression of the PQM-1 transcription factor in the nervous system, which impacts organismal survival to hypoxia (low oxygen) [22]. This unique editing-independent mechanism was uncovered from studies that began with examining mis-regulated genes in neural cells from first-stage larval (L1) animals lacking ADR-2, the sole A-to-I editing enzyme in *C. elegans*. Using a deaminase mutant, the decreased expression of *pqm-1* in *adr-2 (-)* neural cells was determined to be independent of A-to-I editing. Additional biochemical and genetic data indicated that in neural cells lacking *adr-2*, but not wild-type neural cells, ADR-1 binds to *pqm-1* mRNA. Furthermore, disrupting ADR-1 binding in *adr-2 (-)* animals restored neural *pqm-1* levels to wild-type. Interestingly, ADR-1 binding to *pqm-1* was also influenced by nutrients; ADR-1 binding occurred in L1 animals hatched in the absence of food (a standard developmental synchronization method in *C. elegans*) but not when the L1 animals were fed for 6 h after hatching.

The specific regulation of neural *pqm-1* levels to alter organismal survival to hypoxia added to the growing list of environmentally-induced changes in neuronal gene expression that impact behavior [23]. However, it was unclear how this pathway is inhibited in wild-type animals and activated in the absence of *adr-2*. In the present study, high-throughput sequencing approaches were taken to assess the impact of ADR-1 binding on gene expression in the nervous system. Additionally, an unbiased forward genetic screen was performed to identify factors influencing ADR-1-mediated post-transcriptional gene regulation in the nervous system. Our findings indicate the presence of a neural cohort of 43 other genes, like *pqm-1*, which are downregulated upon ADR-1 binding. Further, our data demonstrates that the kinase GSK-3 influences this process in the nervous system.

## Materials and methods

### *C. elegans* strains and maintenance

All worms were maintained under standard laboratory conditions on nematode growth media (NGM) seeded with *Escherichia coli* OP50. The following previously generated strains were used in this study: Bristol strain N2, BB19 (*adr-1 (tm668)*) [24], BB20 (*adr-2*(*ok735*)) [24], BB21 (*adr-1* (*tm668*);*adr-2* (*ok735*)) [24], HAH25 (BB19 + blmEx20 (prab3::GFP::unc-54 3′ UTR (pHH21); prab3::3XFLAG ADR-1::unc-54 3′ UTR (pHH512))) [22], HAH26 (BB21 + blmEx20 (p*rab3*::GFP::*unc-54* 3′ UTR (pHH21); p*rab3*::3XFLAG ADR-1::*unc-54* 3′ UTR (pHH512))) [22], BB76 (p*rab3*::RFP::*C35E7.6* 3′ UTR; p*rab3*::GFP::*unc-54* 3′ UTR; *unc-119* genomic rescue) [24], BB78 (*adr-2* (*ok735*); p*rab3*::RFP::*C35E7.6* 3′ UTR; p*rab3*::GFP::*unc-54* 3′ UTR; *unc-119* genomic rescue) [24], and HAH57 (*adr-2* (*ok735*); 3X FLAG ADR-1 with mutations in dsRBD1 (K223E, K224A, and K227A); p*rab3*::rfp::*C35E7.6* 3′ UTR; p*rab3*::*gfp*::*unc-5*4 3′ UTR; *unc-119* genomic rescue) [22], HAH27 (*adr-2* (*ok735*), agIs6 [*dod-24*::GFP]) [22], HAH28 (BB20 + blmEx18 (*Y75B8A.8* 3′ UTR hairpin construct in p*rab3*::GFP::*unc-54* 3′ UTR (pHH340); p*rab3*::3XFLAG ADR-2 cDNA::*unc-54* 3′ UTR (pHH438) [22], agIs6 [*dod-24*::GFP]) [25], MAH677 (*sid-1 (qt9);rgef-1p*::GFP + *rgef-1p*::*sid-1*) [26], HAH47 (3X FLAG ADR-1 dsRBD1 (K223E, K224A, K227A)) [22], and SD1241 (gaIs153 (pPRSK29 (*F25B3.3*::FLAG::PAB-1);TG96 (*sur-5*::GFP) [27].

Strains generated in this study include HAH59 (3X FLAG ADR-1), HAH66 (*adr-2* (*ok735*), agIs6[*dod-24*::GFP], 3X FLAG ADR-1), HAH67 (*adr-2* (*ok735*);*dsh-2* (*fs*)), HAH68 (BB19 + blmEx20 (p*rab3*::GFP::*unc-54* 3′ UTR (pHH21); p*rab3*::3XFLAG ADR-1::*unc-54* 3′ UTR (pHH512))), HAH69 (BB21 + blmEx20 (p*rab3*::GFP:: *unc-54* 3′ UTR (pHH21); p*rab3*::3XFLAG ADR-1::*unc-54* 3′ UTR (pHH512))), HAH70 (BB21 + blmEx20 (p*rab3*::GFP::*unc-54* 3′ UTR (pHH21); prab3::3XFLAG ADR-1::unc-54 3′ UTR (pHH512)), *dsh-2*(*fs*)), HAH71 (wild-type), HAH72 (adr-2 (ok735)), HAH73 (*adr-2* (*ok735*);*dsh-2* (*blm18*)), HAH74 (*dsh-2* (*blm18*)), HAH75 (3X FLAG ADR-1), HAH76 (3X FLAG ADR-1;*adr-2* (*ok735*)), HAH77 (3X FLAG ADR-1; *sid-1 (qt9);rgef-1p*::GFP + *rgef-1p*::*sid-1*), HAH78 (N2 + 3X FLAG ADR-1), HAH79 (*adr-2* (*ok735*), agIs6[*dod-24*::GFP]; 3X FLAG ADR-1 mutagenized with EMS), HAH80-HAH85 (*adr-2* (*ok735*), agIs6[*dod-24*::GFP]; 3X FLAG ADR-1 mutagenized with EMS).

Animals created by CRISPR modification (HAH67 and HAH78) used standard microinjection techniques and were identified as rolling F1 progeny and nonrolling F2 progeny. Injection mix for HAH67 included 1.5 μM Cas9 (IDT, Alt-R Cas9 nuclease V3), 4 μM tracrRNA, 3 μM of crRNA (HH4324) for *dsh-2*, 1 μM of crRNA (HH3118) for *dpy-10*, and 3 μM of repair template ssODN (HH4325) for *dsh-2* containing the cytidine insertion mutation in *dsh-2* and 1 μM of repair template ssODN (HH2448) for *dpy-10* (Supplementary Table S4). Genomic modifications were verified using Sanger sequencing. The injection mix for HAH78 included 3 μg/μl in-house purified Cas9, 4 μM tracrRNA, 2 μg/μl of *in vitro* transcribed guide RNA (HH2449) for *adr-1*, 3 μM of repair template ssODN (HH2466) containing the 3xFLAG insertion (Supplementary Table S4), 0.45 μg/μl of *in vitro* transcribed guide RNA (HH2447) for *dpy-10*, and 1.2 μM of repair template ssODN (HH2448) for *dpy-10* (Supplementary Table S4). Genomic modifications were verified by Polymerase Chain Reaction (PCR) using *adr-1* flanking forward primer and *adr-1* flanking reverse primer (Supplementary Table S4), followed by Sanger sequencing. Western blotting was performed to verify the 3xFLAG insertion.

Crosses were performed by putting ∼10 males and 1 hermaphrodite on mating plates. Genotyping was performed for the F1 progeny and F2 progeny (primers mentioned in Supplementary Table S4). The specific crosses performed included: creation of HAH59 by crossing HAH78 hermaphrodites to BB21 males, creation of HAH70 by crossing HAH67 hermaphrodites with HAH25 males, creation of HAH74 by crossing HAH67 hermaphrodites with N2 males, creation of HAH77 by crossing MAH677 hermaphrodites with HAH59 males, creation of HAH80–HAH85 by crossing HAH79 hermaphrodites with HAH66 males six times and isolating HAH80, HAH81, and HAH82 which express high GFP and HAH83, HAH84, and HAH85 that express low GFP.

### Bleaching

Synchronized L1 animals were obtained by bleaching with 5 M NaOH and sodium hypochlorite [28]. After the bleach solution was added, animals were incubated on a shaker at 20°C for 7 min. Embryos were washed thrice with 1× M9 buffer (22.0 mM KH_2_PO_4_, 42.3 mM Na_2_HPO_4_, 85.6 mM NaCl, and 1 mM MgSO_4_) and collected by spinning in a tabletop centrifuge before incubating overnight in 1× M9 at 20°C. The next day, hatched L1 animals were spun down and washed thrice with 1× M9. For the GSK-3 kinase activity inhibition experiments, similar to prior work [29], Laduviglusib dissolved in dimethyl sulfoxide (DMSO) (MedChemExpress) was added to a 50 ml of 1× M9 solution at a final concentration of 25 μM during the overnight incubation.

### RNA immunoprecipitation

For all RNA immunoprecipitation (RIP) experiments, synchronized L1 animals were washed with IP buffer (50 mM HEPES [pH 7.4], 70 mM K-acetate, 5 mM Mg-acetate, 0.05% NP-40, and 10% glycerol) containing a mini EDTA-free cOmplete protease inhibitor tablet (Roche) and UV crosslinked (3 J/cm^2^) using the Spectrolinker (Spectronics). Worm pellets were made using liquid nitrogen and stored at −80°C. Frozen pellets were ground on dry ice and centrifuged at maximum speed for 10 min. Protein concentration was measured using Bradford reagent (Sigma) and 50 or 10 μg of lysate was taken for RNA extraction or monitoring protein expression in input samples, respectively. For the neural ADR-1 and neural ADR-2 RIPs, 500 μg lysate was added to 25 μl anti-FLAG magnetic beads (Sigma). After 1 h incubation at 4°C, the beads were washed thrice with wash buffer (0.5 M NaCl, 160 mM Tris–HCl [pH 7.5], 0.1% NP-40, and 0.25% Triton X-100) containing a mini EDTA-free cOmplete protease inhibitor tablet (Roche). A portion of the IP (2/5) was stored in 2× Sodium Dodecyl Sulfate (SDS) loading buffer and used for immunoblotting. The remaining beads were incubated with 1 μl of RNasin [28] and 0.5 μl of 20 mg/ml Proteinase K [30] at 42°C and 1200 rpm for 15 min. The supernatant was then added to 400 μl of TRIzol (Invitrogen) reagent. RNA was isolated, reverse transcribed and quantified as described below. For the neural PAB-1 RIP experiment, 500 μg lysate was added to 50 μl of anti-FLAG magnetic beads. For the neural ADR-2 RIP experiment, as previously described [31], an additional step of pre-clearing the lysates for 1 h at 4°C using protein G beads (Invitrogen) was performed prior to adding the supernatant to the anti-FLAG magnetic beads. 3/5 of the IP was stored in 2× SDS loading buffer and used for immunoblotting.

### Co-immunoprecipitation

For the co-immunoprecipitation (co-IP) of ADR-1 and VIG-1, ADR-1 IP was performed using anti-FLAG magnetic beads (Sigma) as mentioned above for the RIP but without subjecting the animals to UV crosslinking.

### Immunoblotting

Protein lysates were boiled for five minutes and were subjected to SDS–PAGE. The immunoblot was treated with antibodies against FLAG (Sigma, F1804), purified VIG-1 antibody [32] (kind gift from John Kim), phosphoserine/threonine antibody (BD Biosciences Catalog #612549, kind gift from Peter Hollenhorst) or CGH-1 antibody to the peptide—DPKLYVADQQLVDAADETTA [33] (Fisher Scientific). Protein bands were visualized using enhanced chemiluminescent detection SuperSignal West Femto Maximum Sensitivity Substrate [28] (Fisher Scientific) and SuperSignal West Pico PLUS Chemiluminescent Substrate (Fisher Scientific). Unsaturated images were acquired using Image Lab (version 6.1.0 build 7) in the BIO-RAD ChemiDoc MP imaging system.

### RIP sequencing (RIP-seq)

Isolated RNA was subjected to poly(A) selection using magnetic Dynabeads oligo dT (Invitrogen) prior to library preparation with the KAPA stranded RNA-seq library preparation kit (Roche, ref.: 7962169001). Briefly, RNA samples were fragmented into 200–300 bp strands by incubating samples at 94°C for 6 min and were used to synthesize the first and second strands of complementary DNA (cDNA). Adapters (KAPA S1 adapter kit, ref.: 08005770001) were ligated to the cDNA and the libraries were amplified using 14 PCR cycles. Libraries were sequenced (100 nucleotide single end) on an Illumina NextSeq2000 instrument at the Indiana University Center for Genomics and Bioinformatics. 20–50 million reads and 3–4 million reads were obtained for each of the immunoprecipitated samples, and input samples, respectively.

### Bioinformatic analysis of RIP-seq

In brief, 100 bp single-end reads were aligned to the *C. elegans* reference genome ce11 (WS275) using the following STAR (2.7.11a) parameters: [runThreadN 8 outFilterMultimapNmax 1, outFilterScoreMinOverLread .66, outFilterMismatchNmax 10, outFilterMismatchNoverLmax .3]. FeatureCounts (v2.0.1) was used to count the number of mapped reads to Wormbase (WS275) gene annotations using the [-s 2] flag. Genes with read counts of zero across all analyzed samples were eliminated. To identify transcripts enriched in the IP samples of the experimental samples (Neural ADR-1;*adr-2 (-)*) over the control samples (Neural ADR-1), mapped reads for two biological replicates of both IP and input samples were analyzed. Raw read counts were analyzed in R with DESeq2 (v1.26.0) [34] to test for ratio of ratios using a likelihood ratio test [(IP experimental/Input experimental)/(IP control/Input control)].

### RNA isolation and quantitative real-time PCR

RNA extraction was performed using TRIzol (Invitrogen) and DNA contamination was removed by treatment with TURBO DNase (Ambion) followed by either the RNeasy Extraction kit (Qiagen) or the Zymo Clean and Concentrator Kit (Zymo) and stored at −80°C. Concentration and purity of the RNA samples was determined using a Nanodrop (Fisher Scientific). For quantitative real-time PCR (qPCR) experiments using RNA from L1 animals, 1–2 μg of DNase-treated RNA was reverse transcribed into cDNA using Superscript III (Invitrogen) with random hexamer (Fisher Scientific) and oligo dT (Fisher Scientific) primers and 20 μl of water was added to the cDNA. For qPCR of RNA isolated from neural cells and IPs, the entire RNA isolation from the respective samples was reverse transcribed into cDNA and no water was added to the cDNA. For inputs, 500 ng RNA was reverse transcribed into cDNA and no water was added to the cDNA. Gene expression was determined using SybrFast Master Mix water and gene-specific primers (Supplementary Table S4) on a Thermofisher Quantstudio 3 instrument. The primers designed for qPCR (Supplementary Table S4) spanned an exon–exon junction to prevent detection of genomic DNA in the samples. Melting curves were generated for all primer pairs. For each gene, a standard curve of cycle threshold versus the relative concentration of amplified product. of eight to ten samples of 10-fold serial dilutions of the amplified product was generated. Standard curves were plotted on a logarithmic scale in relation to concentration and fit with a linear line. Fit (*r*^2^) values were between 0.98 and 1 and at least seven data points fell within the standard curve. Each cDNA measurement was performed in three technical replicates.

### Neural cell isolation, sequencing, and bioinformatic analysis

Neural cells were isolated from L1 animals as previously described [22]. High-throughput sequencing of poly(A) selected RNA from the isolated neural cells from three independent biological replicates was performed. DESeq2 software [26] was used to assess differential gene expression between *adr-2 (-)* neural cells in the presence and absence of the ADR-1 RNA binding mutant. In brief, 75 bp single-end stranded RNA-sequencing reads were aligned to the *C. elegans* reference genome (WS275) using STAR (v2.7.8a) with the parameters: [runThreadN 8, outFilterMultimapNmax 1, outFilterScoreMinOverLread 0.66, outFilterMismatchNmax 10, outFilterMismatchNoverLmax: 0.3]. Indexing of the aligned bam files was performed using samtools (v1.3.1), and featureCounts (v2.0.1) was used to generate the raw read counts file. DESeq2 library (v1.26.0) on R studio [34] was used for data processing and generating the counts.csv file used for differential gene expression analysis.

### Gene set enrichment analysis and overlaps

Gene set enrichment analysis was performed by entering wormbase IDs into the *C. elegans* specific WormCat software [35] and the *C. elegans* reference genome was used for the background list of genes. The overlap between the RIP-seq and neural RNA-seq datasets was performed using BioVenn [36].

### Editing assays

Total RNA was isolated from mixed-stage animals using TRIzol (Invitrogen) and Zymo Direct-zol RNA miniprep kit (Zymo) with DNase treatment. Two micrograms of RNA was reverse transcribed using Superscript III RT (Invitrogen), and the cDNA was PCR amplified using Phusion High-Fidelity DNA Polymerase [30]. Primers used for reverse transcription and PCR are listed in Supplementary Table S4. PCR products were gel purified and 50 ng of the purified PCR product was subjected to Sanger sequencing. The adenosine and guanosine peak heights were measured on the Photoshop software. % editing was quantified by calculating [(guanosine peak height)/(guanosine peak height + adenosine peak height)]* 100. Negative controls without the Superscript III enzyme were performed to eliminate the possibility of genomic DNA contamination and amplification.

### Ethylmethanesulfonate mutagenesis screen

Bleaching was performed to obtain synchronized L1 animals of strain HAH66. L1 animals were grown for 41 h after hatching to obtain L4 animals. Approximately 2500 L4 animals were exposed to 50 mM ethylmethanesulfonate (EMS) (Sigma–Aldrich) for 4 h and washed thrice with 1× M9 buffer. These animals were transferred to fresh seeded NGM plates and allowed to lay eggs overnight. Next day, animals were washed off using 1× M9 buffer, and the F1 eggs remained on the plates. These F1 eggs were grown to young adults (∼3 days) and the adults were bleached to obtain F2 eggs. The hatched F2 animals were transferred to 15 cm large NGM plates without any bacterial food source and screened for GFP levels under the microscope (Nikon SteREO Discovery microscope). This whole procedure was performed in two technical replicates.

### Mutant Complementation Analysis

Each of the 11 EMS candidates were crossed to each other and GFP levels of the progeny were visualized. If a cross between high GFP candidates produced high GFP progeny, the mutations were considered to be in the same gene; if it produced low GFP progeny, the mutations were considered to be in different genes.

### Backcrossing and isolation of nonmutant siblings

Screen candidates were backcrossed six times with the parental strain used in the mutagenesis. Males (∼10) from the parental strain were crossed with 1 hermaphrodite from the candidates. To screen for high GFP in starved L1 animals specifically, the F1 animals from the mating plate were bleached and examined in the absence of food (starved F2 animals). In the final backcross, three high GFP animals and three low GFP nonmutant sibling animals were obtained and subjected to whole genome sequencing. Variant calling was used to identify single nucleotide differences between the reference genome and the screen candidates (Supplementary Fig. S5). Mutations in nonmutant siblings were subtracted similar to a method previously described [37].

### Whole genome sequencing

Genomic DNA was extracted from the animals using the DNeasy Blood and Tissue Kit (Qiagen).Genomic DNA libraries were made using the NEBNext Ultra II DNA Library Prep with Sample Purification Beads kit [30]. Single end 100 bp reads were aligned to the *C. elegans* reference genome ce11 (WS275) using bwa. The aligned reads were sorted using samtools following which a read pileup was generated and single nucleotide variants were obtained using bcftools mileup. A python script was written to identify single nucleotide variants present in all the high GFP animals but not in low GFP animals. Additionally, known single nucleotide polymorphisms (SNPs) were eliminated from the analysis. The in-depth code that was followed is available on GitHub: https://github.com/ananya716/GSF3874-EMS-pilot/blob/main/Restarting%20from%20alignment

### RNA interference

Gravid adults were bleached to get synchronized L1 animals which were plated on 10-cm RNA interference (RNAi) plates [NGM plates with ampicillin (50 μg/ml), tetracycline (10 μg/ml) and isopropyl β-D-thiogalactopyranoside (IPTG) (2 mM)] seeded with HT115 bacteria containing RNAi vectors against various proteins [38] (Supplementary Table S5). RNAi vectors were sequenced and verified. Animals were also fed with an empty RNAi vector as a control. The L1 animals were grown to gravid adults and bleached again to obtain L1 animals hatched from RNAi-treated animals.

### RNA pulldown

Lysates were precleared using MyOne Streptavidin beads [28] at 37°C for 1 h. After preclearing, 500 pmoles of biotinylated probes (Supplementary Table S4) were incubated with 1000 μg protein lysate and oligo hybridization buffer at 37°C for 2 h. After RNA-probe hybridization, 100 μl MyOne Streptavidin beads were added to the reaction and incubated at 37°C for 1 h. After pulldown, either RNA elution using DNase treatment or protein elution using RNase H treatment was performed. For the second biological replicate for mass spectrometry, two technical replicates were pooled before the elutions. Samples were incubated either at 37°C for 1 h (RNA elution) or 37°C for 3 h with shaking at 1300 rpm (protein elution).

### Mass Spectrometry Data Analysis

On bead digests: After washing, beads were covered with 8 M urea, 100 mM Tris hydrochloride, pH 8.5, reduced with 5 mM tris (2-carboxyethyl) phosphine hydrochloride (TCEP, Sigma–Aldrich Cat. No.: C4706) for 30 min at room temperature. The resulting free cysteine thiols were alkylated using 10 mM choloracetamide (CAA, Sigma–Aldrich Cat. No.: C0267) for 30 min at RT, protected from light. Samples were diluted to 2 M urea with 50 mM Tris pH 8.5 and proteolytic digestion was carried out with Trypsin/LysC Gold (0.4 μg, Mass Spectrometry grade, Promega Corporation Cat. No.: V5072) overnight at 35°C. After digestion, samples were quenched with 0.4% trifluoroacetic acid (v/v, Fluka Cat. No.: 91699). LC-MS/MS: Following quench, samples were desalted on Pierce C18 Spin columns (Cat. No. 89 870) with a wash of 200 μl of 0.5% trifluoroacetic acid and elution in 70% acetonitrile and 0.1% formic acid (FA). After drying peptides in a speed vacuum, samples were resuspended in 25 μl of 0.1% FA. Approximately one-fifth of each sample was injected onto either a 25 cm EasySpray column (ES902 Thermo Fisher Scientific) or a 25 cm IonOpticks column (Ultimate-TS, IonOpticks) using an EasyNano1200 LC coupled to an Exploris 480 orbitrap mass spectrometer (Thermo Fisher Scientific). Solvent B (80% acetonitrile and 0.1% FA) was increased from 8% to 35% over 90 min, increased from 35% to 65% over 15 min, increased to 85% over 5 min, held at 85% for 5 min, and decreased to 4% over 5 min at 300 nl/min. The mass spectrometer was operated in positive ion mode, advanced peak determination on, default charge state of 2 and user defined lock mass of 445.12003. Four-second cycle time was used with MS1 parameters of scan range 375–1500 *m/z*; orbitrap resolution of 120 000, standard AGC, automatic max IT, and RF lens of 40%. Monoisotopic peak determination was set to peptide with a minimum intensity filter of 5.0e3, charge state filter of 2–7, and dynamic exclusion of 30 s with 5 ppm mass tolerance. Three-second top S cycle time was used for fragmentation. MS2 parameters included an isolation window of 1.6 *m/z*, normalized high energy dissociation energy of 30%, orbitrap resolution of 15 000, user defined first mass of 110 *m/z*, standard AGC target and auto max IT. Data were recorded using Tune application 4.2.362.42. Data were analyzed using Proteome Discoverer 2.5.0.400 (Thermo Fisher Scientific). *C. elegans* reference proteome (downloaded from Uniprot on 25 November 2024 with 26 690 sequences and on 13 May 2022 with 78 806 sequences, respectively), plus common laboratory contaminants (73 sequences) was searched using SEQUEST HT. Precursor mass tolerance was set to 10 ppm and fragment mass tolerance set at 0.02 Da with a maximum of three missed cleavages. A maximum of three modifications were allowed per peptide. Percolator false discovery rate (FDR) filtration of 1% was applied to both the peptide-spectrum match and protein levels. Search results were loaded into Scaffold Q + S Software (version 5.2.2, Proteome Software, Inc.) for visualization.

### Statistical analysis

GraphPad Prism recommended statistical sets were performed and are indicated in the figure legends.

## Results

### ADR-1 mediates neural gene expression of a unique regulon

In our previous work, we demonstrated that ADR-1 binds to the *pqm-1* transcript in *adr-2 (-)* L1 neural cells, leading to downregulation of the transcription factor PQM-1, which plays a crucial role in the organism's response to hypoxia [22]. We sought to determine if binding and downregulation by ADR-1 was specific to *pqm-1* or if this post-transcriptional gene regulation occurred extensively in the nervous system. To achieve this goal, transcripts bound by ADR-1 in the nervous system in the presence and absence of *adr-2* were identified in a transcriptome-wide manner.

**Figure 1.**
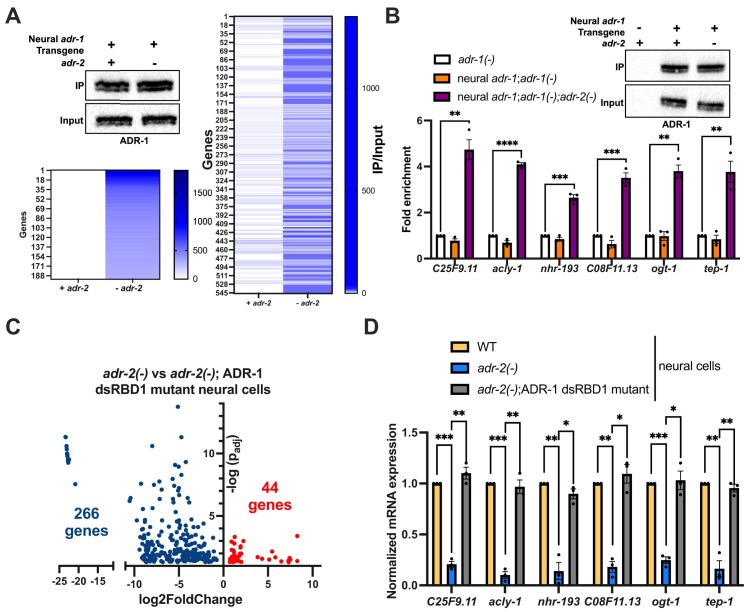
ADR-1 binding controls gene expression of a neural regulon in *adr-2(-)* animals. (**A**) Top left: Lysates and immunoprecipitates from the indicated strains were subjected to immunoblotting with a FLAG antibody. Blot is a representative image from two independent biological replicates. Bottom left: Neural ADR-1 bound targets only in *adr-2(-)*. Right: Heat map of the average raw read counts of the immunoprecipitated sample divided by those from the input sample were plotted with each line representing a gene. A darker color indicates higher read counts of the gene in the immunoprecipitate compared to input, white color indicates read count of 0 in immunoprecipitate. (**B**) Lysates and immunoprecipitates from the indicated strains were subjected to immunoblotting with a FLAG antibody. Blot is a representative image from three independent biological replicates. Bar graph represents the fold enrichment of cDNA of indicated genes in the IP samples relative to the amount of cDNA in the input lysate for each strain. The IP/input values are obtained for each strain and then normalized to the IP/input value for *adr-1(-)* animals. The mean of three independent biological replicates was plotted. Error bars represent SEM. Statistical significance was calculated using multiple unpaired *t* tests followed by Holm–Šídák multiple comparisons correction. **P < 0.005, ***P< 0.0005, ****P< 0.0001. (**C**) Volcano plot depicting genes with significantly decreased (blue dots, *P*_adj_ < 0.05 and log2foldchange < −0.5) or increased (red dots, *P*_adj_< 0.05 and log2foldchange > 0.5) expression in RNA-seq datasets from *adr-2(-)*;ADR-1 dsRBD1 mutant neural cells compared to *adr-2(-)* neural cells. log2foldchange is plotted on the *X-*axis and −log(*p_adj_*) value is plotted on the *Y-*axis. (**D**) Expression of the indicated genes was determined relative to expression of the housekeeping gene *gpd-3* in isolated neural cells from the strains indicated. Values were then normalized to WT neural cells and the average of three biological replicates was plotted. Statistical significance was calculated using multiple unpaired *t* tests followed by Holm–Šídák multiple comparisons correction and the error bars represent SEM; *P < 0.05, **P < 0.005, ***P < 0.0005. For panel (A), the indicated genotypes are of strains HAH25 and HAH26. For panel (B), the indicated genotypes are of strains BB19, HAH25 and HAH26. For panel (C), the indicated genotypes are strains BB78 and HAH57. For panel (D), the indicated genotypes are strains BB76, BB78, and HAH57.

ADR-1 and associated RNAs were immunoprecipitated from wild-type and *adr-2 (-)* animals expressing ADR-1 solely in the nervous system, as we have done previously [22]. After confirming a successful ADR-1 immunoprecipitation (Fig. 1A), neural ADR-1 associated RNAs were isolated and subjected to high-throughput sequencing. To control for differential gene expression between wild-type and *adr-2 (-)* animals, sequencing was also performed on RNA isolated from the lysates prior to immunoprecipitation. The raw read counts from two independent biological replicates were analyzed with DESeq2 with the likelihood ratio test [34] to identify neural ADR-1 bound RNAs. Through this analysis, 545 transcripts either bound significantly more by ADR-1 in the absence of *adr-2* or uniquely bound by ADR-1 in the absence of *adr-2* were identified in the nervous system (log_2_foldchange ≥ 1, *P*_adj_*<*0.001, Supplementary Table S1).

Seeking to identify transcripts regulated similarly to *pqm-1*, only genes that exhibited no reads in the ADR-1 IPs from wild-type animals were considered further (Fig. 1A and Supplementary Table S1). From this analysis, 193 genes were identified as uniquely bound by ADR-1 in the absence of *adr-*2, and as expected, *pqm-1* was one of these 193 transcripts.

To specifically test that these genes are bound by neural ADR-1 only in the absence of *adr-2*, neural ADR-1 binding to six randomly selected genes was examined using an RNA immunoprecipitation assay coupled to qPCR (RIP qPCR). As expected, neural ADR-1 was immunoprecipitated in the presence and absence of *adr-2* but not from the negative control animals lacking *adr-1* (Fig. 1B). Importantly, when compared to the animals lacking *adr-1*, a significant enrichment of all six transcripts was observed in the neural ADR-1 IPs in the absence of *adr-2* (Fig. 1B). However, there was no significant enrichment in the neural ADR-1 IPs in the presence of *adr-2* (Fig. 1B). These data demonstrate that these transcripts are uniquely bound by ADR-1 in the nervous system only in the absence of *adr-2*.

Next, we sought to determine whether ADR-1 binding to these transcripts altered gene expression in neural cells. To test this, neural cells were isolated from *adr-2 (-)* animals with wild-type ADR-1 and *adr-2 (-)* animals that express a mutant of ADR-1 that lacks the ability to bind RNA. The ADR-1 RNA binding mutant has mutations within the conserved KKxxK (where K is lysine, and x is any amino acid) motif in the first dsRNA-binding domain (dsRBD1), which was previously shown to disrupt the ability of ADR-1 to bind RNA *in vivo* [22, 39].

High-throughput sequencing from three independent biological replicates was performed. Differential gene expression analysis identified 310 transcripts that were significantly mis-expressed in neural cells from *adr-2 (-)* animals expressing the ADR-1 RNA binding mutant compared to neural cells from *adr-2 (-)* animals expressing wild-type ADR-1 (Fig. 1C and Supplementary Table S2). Out of the 310 transcripts identified, 266 transcripts had decreased neural expression, and 44 transcripts had increased neural expression in the absence of ADR-1 binding in *adr-2 (-)* animals (Fig. 1C and Supplementary Table S2).

To specifically determine if the misexpression is due to direct binding by neural ADR-1, all 310 mis-expressed transcripts were overlapped with the 193 transcripts uniquely bound by ADR-1 in the absence of *adr-2* (Fig. 1A). Only 6/266 downregulated transcripts were bound by ADR-1 in the absence of *adr-2*, suggesting that many of the downregulated transcripts may be indirect targets of ADR-1. Alternatively, it is possible that these genes are bound by ADR-1 but not differentially regulated in the absence of *adr-2*, which our experiments cannot address due to the lack of ADR-1 RIP data from *adr-1 (-)* animals. In contrast, all 44 upregulated transcripts were uniquely bound by neural ADR-1 in the absence of *adr-2* (Supplementary Fig. S1). As expected, *pqm-1* was one of the 44 transcripts identified in this analysis.

Thus far, our data indicate that 44 transcripts which are all bound by ADR-1, exhibit increased neural expression upon loss of ADR-1 binding (Fig. 1C). However, it is unclear if, similar to *pqm-1*, these transcripts are downregulated in *adr-2 (-)* neural cells when compared to wild-type. To test this, qPCR was performed for the six previously selected genes in neural cells isolated from wild-type and *adr-2 (-)* animals in the presence and absence of ADR-1 RNA binding. Compared to wild-type neural cells, all six transcripts had significantly decreased expression in *adr-2 (-)* neural cells (Fig. 1D). As observed in the neural RNA sequencing data (Fig. 1C), all six transcripts examined were significantly upregulated in neural cells from *adr-2 (-)* animals lacking ADR-1 binding compared to *adr-2 (-)* neural cells (Fig. 1D). These results suggest that in the absence of *adr-*2, ADR-1 RNA binding downregulates the expression of a cohort of neural transcripts.

To determine if the neural post-transcriptional regulation by ADR-1 impacted specific biological processes, gene set enrichment analysis was performed using WormCat [35]. This *C. elegans* specific software revealed a significant enrichment for genes involved in lipid metabolism (28/44 genes) within the neural regulon genes that are upregulated in *adr-2 (-)*;ADR-1 dsRBD1 mutant neural cells compared to *adr-2 (-)* neural cells (Supplementary Fig. S2 and Fig. 1D). Although prior work from others had indicated that *pqm-1* is important for lipid metabolism [40], only one of these genes is a known PQM-1 target. In contrast, a similar analysis of the downregulated genes did not reveal an enrichment for lipid metabolism genes (Supplementary Fig. S2). Interestingly, global modulation of lipid synthesis has been shown to contribute to stress resistance and longevity in *C. elegans* [41]. Our previous work has already demonstrated that ADR-1-mediated neural downregulation of *pqm-1* altered organismal survival to hypoxia [22]. Beyond *pqm-1*, global downregulation of other cohort genes, including *hyl-1*, *lipl-5, maoc-1*, and *fat-3*, has been linked to increased survival under heat and oxidative stress [42], resistance from anoxia [43], survival to food deprivation [44], and increased longevity [45, 46].

Together, our findings indicate that RNA binding by ADR-1 contributes to downregulation of transcripts involved in lipid metabolism in neural cells, which may play an important role in stress resistance and longevity.

### A forward genetic screen to identify regulators of neural ADR-1 binding

Thus far, our findings demonstrate the presence of a unique neural regulon that is post-transcriptionally regulated by ADR-1 binding in *adr-2 (-)* animals. Since ADR-1 binding occurs only in the absence of *adr-2*, it is possible that ADR-2 binds or edits these transcripts to prevent ADR-1 from binding. However, our previous work demonstrated that ADR-1 binding and downregulation of neural *pqm-1* is independent of RNA editing by ADR-2 [22]. To assess the possibility that ADR-2 binds transcripts of the neural regulation and blocks ADR-1 binding in the wild-type nervous system, ADR-2 was immunoprecipitated from animals that solely express ADR-2 in the nervous system and the presence of regulon genes was quantified using RIP qPCR. Compared to *adr-2 (-)* animals, neural ADR-2 IPs exhibited a significant enrichment of *lam-2*, a known ADR-2 bound gene [31, 39] (Supplementary Fig. S3). In contrast, there was no enrichment for any of the four regulon genes in the neural ADR-2 IPs (Supplementary Fig. S3). These results suggest that ADR-2 does not bind the neural regulon transcripts to prevent ADR-1 from binding. Further, these results suggest that some cellular environment or factor outside of ADR-2 leads to ADR-1 binding to the regulon. To explore this possibility, a forward genetic screen was performed (Supplementary Fig. S4).

EMS mutagenesis was performed on animals containing a GFP transcriptional reporter for the PQM-1 activated gene *dod-24*. The expression of *dod-24* was used as a proxy measurement for the activity of ADR-1 on the neural regulon. We previously demonstrated that *pqm-1* levels are low in these animals due to ADR-1 binding, which in turn results in decreased transcriptional activity and thus, low GFP expression [22]. These animals also contain a genomically engineered 3× FLAG at the N-terminus of *adr-1*, allowing us to measure ADR-1 protein expression in the candidates. The presence of the 3× FLAG tag did not affect the ability of ADR-1 to regulate ADR-2 editing activity (Supplementary Fig. S5). From the progeny of the EMS treated animals, 43 candidates with high GFP expression and presumably altered ADR-1-mediated post-transcriptional regulation were identified. Western blot analysis indicated that 32 of the 43 candidates did not express ADR-1 protein. Since these candidates exhibited altered GFP expression due to lack of ADR-1 itself, these candidates were not pursued further (Supplementary Fig. S5A).

**Figure 2.**
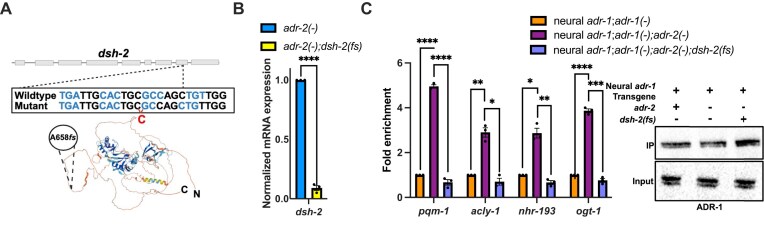
*dishevelled-2 (dsh-2)* regulates ADR-1 binding to the neural regulon in *adr-2(-)* animals. (**A**) Schematic of the predicted AlphaFold structure (Uniprot) of DSH-2 with the A658*fs* mutation mapped. The gray boxes denote *dsh-2* exons and the wild-type and mutant *dsh-2* sequences are indicated. The corresponding wild-type and mutant sequences of ∼10 nucleotides surrounding the insertion are shown, with codons colored in alternating light blue and black. The cytidine insertion in the *dsh-2(fs)* mutant is depicted in red. (**B**) Gene expression measured by qPCR in L1 animals of indicated strains. Expression of *dsh-2* was determined relative to the housekeeping gene *gpd-3*. Obtained values were normalized to *adr-2(-)* animals and an average of three biological replicates was plotted. Statistical significance was calculated using multiple unpaired *t* tests followed by Holm–Šídák multiple comparisons correction and the error bars represent SEM; ****P < 0.0001. (**C**) Right: Lysates and immunoprecipitates from the indicated strains were subjected to immunoblotting with a FLAG antibody. Blot is a representative image from three independent biological replicates. Left: The fold enrichment of cDNA of indicated genes in the IP samples compared to the input samples for each strain is calculated. The IP/input values were obtained for each strain and normalized to the calculated value for the negative control (neural *adr-1;adr-1(-)*) animals. The mean of three biological replicates was plotted. Statistical significance was calculated by multiple unpaired *t* tests followed by Holm–Šídák multiple comparisons correction and the error bars represent SEM; *P < 0.05, **P < 0.005, ***P < 0.0005, and ****P < 0.0001. For panel (B), the indicated genotypes are strains HAH72 and HAH73. For panel (C), the indicated genotypes are strains HAH68, HAH69, and HAH70.

After performing mutant complementation tests on the remaining 11 candidates from the EMS screen, five different complementation groups were obtained (Supplementary Fig. S5B). The complementation group with three candidates from the screen was chosen for whole genome sequencing (Supplementary Fig. S6). A single nucleotide (cytidine) insertion in the coding sequence of the *DISHevelled related* gene (*dsh-2*) was the only common mutation in the sequenced complementation group which was absent in the nonmutant siblings (Fig. 2A). The insertion in *dsh-2* is predicted to disrupt the reading frame of DSH-2, altering 33 amino acids after Ala658 and ultimately generating a premature stop codon (referred to hereafter as *dsh-2 (fs)*). On the DSH-2 structure generated by AlphaFold, the Ala658 and subsequent amino acid changes are expected to lie in a disordered region (Fig. 2A).

It is likely that the premature stop codon impacts *dsh-2* mRNA levels via nonsense mediated decay. To directly test this possibility, CRISPR genome engineering was used to specifically generate the *dsh-2 (fs)* mutation in a clean *adr-2 (-)* genetic background. Compared to *adr-2 (-)* animals, there was a significant reduction in *dsh-2* mRNA expression in the *adr-2 (-);dsh-2 (fs)* animals (Fig. 2B). These results suggest that the *dsh-2 (fs)* mutation is a loss of function mutation of *dsh-2*.

To confirm that the *dsh-2 (fs)* mutation was the specific genomic mutation within the EMS isolated candidate leading to altered ADR-1 binding, neural ADR-1 binding to several members of the regulon identified above was measured using RIP qPCR. From western blot analysis, ADR-1 was successfully immunoprecipitated from all strains (Fig. 2C). As expected, there was a significant enrichment of all transcripts of the regulon in the neural ADR-1 IP samples from animals lacking *adr-2* (Fig. 2C). Importantly, compared to the neural ADR-1 IP samples from animals lacking *adr-*2, the presence of the *dsh-2 (fs)* mutation significantly reduced binding of neural ADR-1 to all transcripts of the regulon examined (Fig. 2C). These observations suggest that DSH-2 activity is required in *adr-2 (-)* animals for ADR-1 to bind to the neural regulon.

### The kinase GSK-3 inhibits ADR-1 binding to the regulon independent of the WNT pathway

As DSH-2 is a signal transducer in the WNT pathway [47], it is possible that ADR-1 binding is regulated by DSH-2 through downstream effectors of WNT signaling. Some immediate downstream effectors include the APC ortholog *apr-1* and the kinases *kin-19* and *gsk-3* [48]. The activity of these WNT effectors is inhibited by DSH-2 [48]. Thus, since *dsh-2 (fs)* is a loss of function mutation, it would be expected that activation of these downstream proteins results in loss of ADR-1 binding.

**Figure 3.**
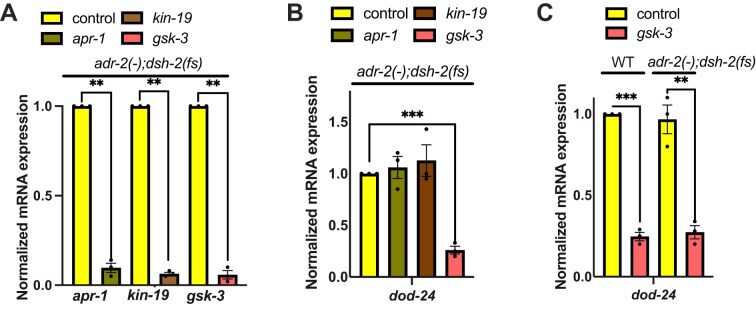
*dsh-2* regulates ADR-1 binding through the kinase GSK-3 in *adr-2(-)* animals. (**A–** **C**) Gene expression of L1 animals measured by qPCR after treatment with RNAi against indicated genes. Expression of indicated genes was determined relative to expression of the housekeeping gene *gpd-3*. Values were then normalized to (A and B) *adr-2(-);dsh-2(fs)* + control RNAi animals or (**C**) WT + control RNAi animals. The bar graphs represent the mean of three biological replicates. Statistical significance was calculated using multiple unpaired *t* tests followed by Holm–Šídák multiple comparisons correction and the error bars represent SEM; **P < 0.005, ***P < 0.0005. For panel (A and B), the indicated genotype is strain HAH73. For panel (C), the indicated genotypes are strains HAH71 and HAH73.

To determine the impact of each of these WNT effectors, each effector was individually reduced using RNAi and expression of *dod-24* was monitored as a readout of ADR-1 binding to *pqm-1*. Reduced expression of each WNT effector was confirmed for the RNAi treatments (Fig. 3A).

Compared to control animals, there was no significant difference in *dod-24* expression when animals were treated with *apr-1* RNAi or *kin-19* RNAi (Fig. 3B). However, *adr-2 (-);dsh-2 (fs)* animals treated with RNAi against *gsk-3* exhibited significantly decreased *dod-24* expression (Fig. 3B). This suggests that DSH-2 impacts neural ADR-1 binding to *pqm-1*, and potentially other members of the regulon, through GSK-3. Additionally, since APR-1, KIN-19, and GSK-3 function together as a complex in the WNT pathway, these results also suggest that ADR-1 binding is regulated independently of the transcriptional output of the WNT pathway.

So far, our results suggest that loss of GSK-3 alters post-transcriptional gene regulation by ADR-1 in *adr-2 (-);dsh-2 (fs)* animals. To determine whether loss of GSK-3 alone is sufficient to regulate ADR-1 function in wild-type animals, *dod-24* expression was measured in wild-type animals in the presence and absence of *gsk-3*. Interestingly, wild-type animals treated with RNAi against *gsk-3* exhibited significantly decreased *dod-24* expression (Fig. 3C). These results indicate that global loss of GSK-3 in wild-type animals likely influences neural ADR-1 binding.

### Presence of GSK-3 in the nervous system inhibits ADR-1 binding to the regulon

**Figure 4.**
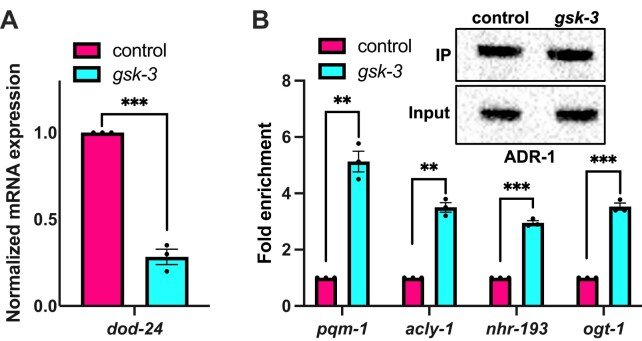
GSK-3 in the nervous system inhibits ADR-1 binding to the regulon. (**A**) Gene expression of L1 animals measured by qPCR after treatment with RNAi against indicated genes specifically in neural cells. Expression of the indicated gene was determined relative to expression of the housekeeping gene *gpd-3*. Obtained values were normalized to animals treated with control RNAi in neural cells and the mean of three biological replicates was plotted. Statistical significance was calculated using multiple unpaired *t* tests followed by Holm–Šídák multiple comparisons correction and the error bars represent SEM; ***P < 0.0005. (**B**) Lysates and immunoprecipitates from animals treated with RNAi in neural cells against the indicated genes were subjected to immunoblotting with a FLAG antibody. Blot is a representative image from three independent biological replicates. Plotted bar graphs represent the fold enrichment of cDNA of the indicated genes in the IP samples compared to the input samples from animals treated with indicated RNAi conditions. The IP/input values were normalized to the calculated value for control neuronal RNAi animals. The mean of three biological replicates was plotted. Statistical significance was calculated using multiple unpaired *t* tests followed by Holm–Šídák multiple comparisons correction and the error bars represent SEM; **P < 0.005, ***P < 0.0005. For all panels of this figure, the indicated genotype is strain HAH77.

Since previous work indicated that changes to ADR-1 binding and *pqm-1* levels specifically in neural cells impacted *dod-24* expression in the whole animal [22], we sought to directly test whether GSK-3 function in the nervous system is important in this regulatory mechanism. To do this, *dod-24* expression was measured upon *gsk-3* RNAi treatment of animals in which RNAi occurs only in the nervous system [26]. Treating the neuronal RNAi animals with RNAi against *gsk-3* resulted in a significant reduction in *dod-24* expression (Fig. 4A), suggesting that GSK-3 in the nervous system is sufficient to regulate *dod-24* levels.

Since *dod-24* expression is a proxy for ADR-1 binding to *pqm-1*, these results imply that GSK-3 function in the nervous system alters ADR-1 binding. To directly test this hypothesis, ADR-1 binding was measured using RIP qPCR in the presence and absence of RNAi against GSK-3 in the nervous system. Western blot analysis indicated that ADR-1 was immunoprecipitated similarly from all animals (Fig. 4B). Importantly, compared to control animals, the ADR-1 IP samples from animals treated with *gsk-3* RNAi in the nervous system had a significant enrichment of all regulon genes examined (Fig. 4B). These results suggest that GSK-3 is a novel regulator of ADR-1 binding in the nervous system.

It is possible that GSK-3 influences ADR-1 binding generally, rather than specifically to transcripts of the regulon. To assess this possibility, ADR-1 binding to *lam-2*, a transcript that is not influenced by the presence or absence of *adr-2* [49], was measured in the presence and absence of *gsk-3* RNAi in neural cells. As expected, there was a significant enrichment of *lam-2* in the ADR-1 IP samples in animals treated with control RNAi in neural cells when compared to *adr-1 (-)* animals (Supplementary Fig. S7). In contrast to the regulon genes, ADR-1 binding to *lam-2* was observed in animals treated with *gsk-3* RNAi in neural cells (Supplementary Fig. S7).

Together, these data indicate that GSK-3 regulates ADR-1 binding specifically to transcripts of the neural regulon, not generally inhibiting ADR-1 to binding to RNA.

### Binding of VIG-1 and ADR-1 to the neural regulon is mutually dependent

**Figure 5.**
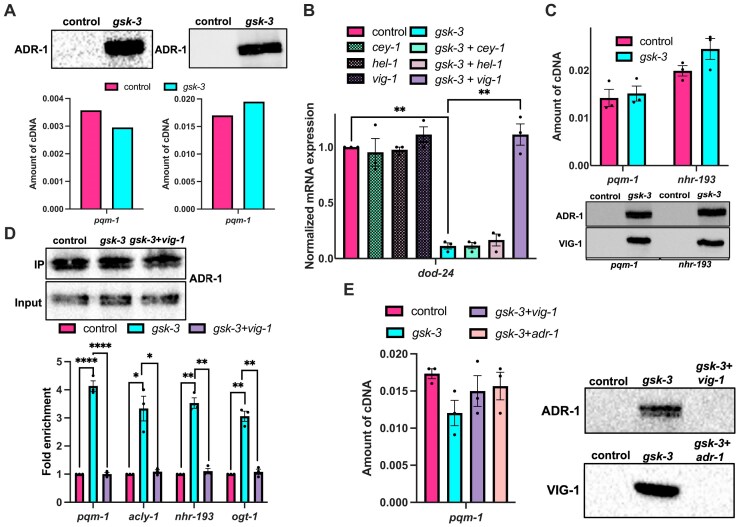
GSK-3 inhibits ADR-1 binding to the regulon through VIG-1. (**A**) Bottom: The cDNA levels of *pqm-1* in the *pqm-1* pulldown (PD) samples from animals after treatment with RNAi against indicated genes specifically in neural cells. Top: *pqm-1* pulldown samples from control and *gsk-3* neuronal RNAi animals were subjected to immunoblotting with a FLAG antibody. (**B**) Gene expression of L1 animals measured by qPCR after treating animals with RNAi against indicated genes. Expression of *dod-24* was determined relative to expression of the housekeeping gene *gpd-3*. Values were normalized to control neuronal RNAi treated animals. The bar graph represents the mean of three biological replicates. Statistical significance was calculated using multiple unpaired *t* tests followed by Holm–Šídák multiple comparisons correction and the error bars represent SEM; **P < 0.001 (**C**) Top- The cDNA levels of *pqm-1* and *nhr-193* in the respective pulldown samples from control and *gsk-3* neuronal RNAi treated animals. Bottom: *pqm-1* and *nhr-193* pulldown samples from control and *gsk-3* neuronal RNAi treated animals were subjected to immunoblotting with a FLAG antibody and VIG-1 antibody. Blot is a representative image from three independent biological replicates. Error error bars represent SEM. (**D**) Bottom: Plotted bar graphs represent the fold enrichment of cDNA of the indicated genes in the IP samples compared to the input samples from animals treated with the indicated RNAi conditions. The IP/input values were obtained for each condition and normalized to the calculated value for the control neuronal RNAi animals. The mean of three biological replicates was plotted. Statistical significance was calculated by multiple unpaired *t* tests followed by Holm–Šídák multiple comparisons correction and the error bars represent SEM; *P < 0.05, **P < 0.005, ****P < 0.0001. Top: Lysates and immunoprecipitates from the animals treated with the indicated RNAi conditions were subjected to immunoblotting with a FLAG antibody. Blot is a representative image from three independent biological replicates. (**E**) Left: Bar graph represents the cDNA levels of *pqm-1* in the *pqm-1* pulldown samples from animals treated with RNAi against indicated genes specifically in neural cells. Error bars represent SEM. Right: Pulldown samples from left panel subjected to immunoblotting with a FLAG antibody (ADR-1) or VIG-1 antibody. Blot is a representative image from three independent biological replicates. For all panels of this figure, the indicated genotype is strain HAH77.

So far, our results demonstrate that GSK-3 specifically inhibits ADR-1 binding to the neural regulon but not generally to mRNA. The mechanism through which this occurs, potentially involves another RBP, which imparts ADR-1 binding specificity, and is associated with the regulon in a GSK-3-dependent manner. To test this possibility, proteins associated with the *pqm-1* transcript were isolated from animals treated with control or *gsk-3* RNAi in the nervous system. To stabilize RNA-protein interactions, animals were subjected to UV crosslinking prior to lysis. The *pqm-1* transcript was pulled down using biotinylated DNA oligos antisense to the *pqm-1* transcript (Fig. 5A, two independent biological replicates). Successful isolation of proteins associated with the *pqm-1* transcript was confirmed by immunoblotting for ADR-1. As expected, ADR-1 was detected in the *pqm-1* pulldown from the animals treated with *gsk-3* RNAi but not animals treated with control RNAi in neural cells (Fig. 5A).

To perform an unbiased assessment of proteins bound to *pqm-1*, the associated proteins were subjected to mass spectrometry. For the *pqm-1* pulldown samples from control animals, 40 proteins and 604 proteins were identified in the first and second replicate, respectively (Supplementary Table S3, tab 2 and tab 4). For the *pqm-1* pulldown samples from *gsk-3* neuronal RNAi animals, 100 proteins and 2168 proteins were identified in the first and second replicate, respectively (Supplementary Table S3, tab 2 and tab 4). The increase in proteins identified in the second mass spectrometry analysis is likely due to the higher amount of *pqm-1* pulled down in the second biological replicate compared to the first (Fig. 5A). Additionally, compared to the control, ∼2–3 times proteins were detected in the mass spectrometry analysis of the *pqm-1* pulldown performed from animals treated with *gsk-3* RNAi in neural cells. This result suggests that loss of *gsk-3* in neural cells can vastly alter the proteins associated with the *pqm-1* transcript.

To identify proteins differentially associated with *pqm-1* in a GSK-3-dependent manner, proteins with a two-fold difference in total number of identified peptides between the control and *gsk-3* neuronal RNAi samples in both biological replicates were considered. From this analysis, a total of 47 proteins were identified to be differentially associated with *pqm-1* in a GSK-3-dependent manner (Supplementary Table S3, tab 5). To focus on the hypothesis that another RBP provides target specificity to ADR-1 in a GSK-3-dependent manner, RBPs differentially identified in the pulldown experiments were selected. From this analysis, three RBPs, CEY-1, HEL-1, and VIG-1, were determined to be observed >2-fold more in *pqm-1* pulldowns from animals treated with *gsk-3* RNAi in neurons compared to control RNAi (Supplementary Table S3, tab 6).

To test whether any of the RBPs identified facilitate ADR-1 binding to the regulon, each of these RBPs were knocked down individually, and *dod-24* expression was measured as a proxy of ADR-1 binding to *pqm-1*. As expected, due to the lack of ADR-1 binding to *pqm-1* in wild-type neural cells (Fig. 2C), there was no impact on *dod-24* expression upon neuronal RNAi treatment of *cey-1*, *hel-1*, or *vig-1* in wild-type animals (Fig. 5B, checkered bars). However, as observed previously (Fig. 4A), there was a significant reduction in *dod-24* expression in wild-type animals treated with *gsk-3* RNAi in neural cells compared to the control (Fig. 5B, magenta bar to teal bar). These data suggest that while loss of GSK-3 can impact ADR-1 RNA binding, loss of CEY-1, HEL-1, or VIG-1 is not sufficient to promote ADR-1 binding in wild-type animals.

However, as the mass spectrometry suggests that these RBPs are present on the *pqm-1* transcript in a GSK-3-dependent manner, the RBPs identified may influence ADR-1 binding to the regulon specifically in the absence of *gsk-3*. To test this possibility, animals treated with *gsk-3* neuronal RNAi were also treated with RNAi of *cey-1*, *hel-1*, or *vig-1*. RNAi against *cey-1* and *hel-1* in animals treated with *gsk-3* RNAi did not significantly affect *dod-24* expression (Fig. 5B). In contrast, animals with treated with RNAi against *gsk-3* and *vig-1* in neural cells exhibited a significant increase in *dod-24* expression (Fig. 5B, teal bar to purple bar). These results suggest that VIG-1 promotes ADR-1 binding to *pqm-1* in a GSK-3-dependent manner.

VIG-1 is expressed in the *C. elegans* nervous system and is abundant in larval stages [50]. To test if VIG-1 binds the regulon genes, two members of the regulon, *pqm-1* and *nhr-193*, were separately pulled down from lysates of synchronized L1 animals treated with control or *gsk-3* RNAi in neural cells (Fig. 5C). As a positive control, the presence of ADR-1 in the pulldowns was detected by immunoblotting (Fig. 5C). Strikingly and similar to ADR-1, VIG-1 was present in the *pqm-1* and *nhr-193* pulldown samples in the animals subjected to *gsk-3* RNAi in neural cells (Fig. 5C), but not in the control animals. These results demonstrate that both VIG-1 and ADR-1 bind mRNAs of the regulon in the absence of GSK-3.

Based on our current findings, we hypothesize that VIG-1 facilitates ADR-1 binding to the neural regulon in the absence of *gsk-3*. To test this hypothesis, ADR-1 binding to the regulon was measured using RIP qPCR. Similar levels of ADR-1 were immunoprecipitated from all animals (Fig. 5D). As observed previously (Fig. 4B), there was a significant enrichment for all four genes of the regulon examined in the ADR-1 IP samples from animals treated with *gsk-3* RNAi in neural cells compared to the control (Fig. 5D). However, compared to animals treated with *gsk-3* neural RNAi alone, animals treated with both *gsk-3* and *vig-1* RNAi in neural cells had significantly decreased ADR-1 binding to the regulon (Fig. 5D). Importantly, *gsk-3* and *vig-1* RNAi did not impact ADR-1 binding to a nonregulon transcript, *lam-2* (Supplementary Fig. S8). Together, these results indicate that loss of VIG-1 abolishes ADR-1 binding to the neural regulon in a GSK-3-dependent manner.

While the results thus far suggest that VIG-1 binding promotes ADR-1 binding to the regulon, it is possible that ADR-1 also promotes VIG-1 binding to the regulon. To test the dependence of VIG-1 RNA binding on the presence of ADR-1, *pqm-1* mRNA was pulled down from lysates of synchronized L1 animals treated with control, *gsk-3* RNAi, and *gsk-3* + *adr-1* RNAi in neural cells (Fig. 5E). Consistent with the RIP qPCR data (Fig. 5D), ADR-1 was present on the *pqm-1* transcript in animals subjected to *gsk-3* RNAi in neural cells but not in animals subjected to both *vig-1* and *gsk-3* RNAi in neural cells (Fig. 5E). As expected, and shown previously (Fig. 5C), VIG-1 was present in the *pqm-1* pulldown samples in the animals subjected to *gsk-3* RNAi in neural cells, but not in the control animals (Fig. 5E). Interestingly, VIG-1 was not present in the pulldown samples upon knocking down both *gsk-3* and *adr-1* in neural cells (Fig. 5E). Together, these results demonstrate that binding of VIG-1 and ADR-1 to the neural regulon is mutually dependent.

### GSK-3 phosphorylation of VIG-1 inhibits VIG-1–ADR-1 complex binding to regulon

**Figure 6.**
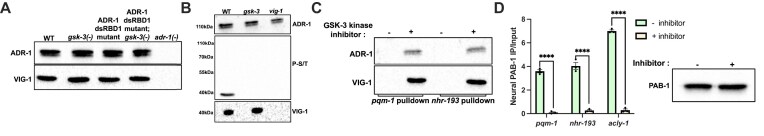
GSK-3 phosphorylation of VIG-1 prevents VIG-1 from binding to regulon. (**A**) FLAG IP samples from animals treated with RNAi against indicated genes were subjected to immunoblotting with FLAG antibody (ADR-1) or VIG-1 antibody. Blot is a representative image from three independent biological replicates. (**B**) FLAG IP samples from animals treated with RNAi against indicated genes were subjected to immunoblotting with FLAG antibody (ADR-1) (top blot), a phosphoserine/threonine antibody (middle blot) or VIG-1 antibody (bottom blot). Blot is a representative image from two independent biological replicates. (**C**) *pqm-1* and *nhr-193* pulldown samples in the presence and absence of Laduviglusib were subjected to immunoblotting with a FLAG antibody (ADR-1) or VIG-1 antibody. Blot is a representative image from three independent biological replicates. (**D**) Bar graph represents the neural PAB-1 IP/input values of cDNA of the indicated genes in the presence and absence of Laduviglusib. The mean of three biological replicates was plotted. Statistical significance was calculated by multiple unpaired *t* tests followed by Holm–Šídák multiple comparisons correction and the error bars represent SEM; ****P < 0.0001. Right: Blot is a representative image from three independent biological replicates. For panel (A), the indicated genotypes are strains HAH59 and HAH47. For panels (B and C), the indicated genotype is strain HAH47. For panel (D), the indicated genotype is strain SD1241.

Our results demonstrate that the absence of either VIG-1 or ADR-1 leads to the loss of the other protein on the neural regulon transcripts. Thus, the mere presence of either RBP cannot be determining the fate of the ADR-1–VIG-1 complex binding to the neural regulon. Based on the genetic screen and prior results, we hypothesized that GSK-3 could influence ADR-1–VIG-1 binding to the regulon either by impacting the ADR-1–VIG-1 interaction or RNA binding of the RBPs. To test these possibilities, we first sought to determine if VIG-1 and ADR-1 physically interact and if so, whether GSK-3 impacted the protein-protein interaction. To gain insight into the ADR-1–VIG-1 interaction, ADR-1 was immunoprecipitated and the presence of VIG-1 was examined. Interestingly, VIG-1 was present in ADR-1 IPs from wild-type animals (Fig. 6A, first lane), where neither protein is bound to the neural regulon (Fig. 5C–E). VIG-1 was also present in the ADR-1 IPs from animals treated with RNAi to *gsk-3* (Fig. 6A, second lane). These results suggest that ADR-1 and VIG-1 physically interact, and GSK-3 does not impact this interaction.

While the above data suggest that VIG-1 and ADR-1 physically interact, as both proteins are capable of binding RNA, and we have demonstrated that the two RBPs bind the same targets *in vivo* (Fig. 5), it is possible that the ADR-1–VIG-1 co-immunoprecipitation observed is due to both RBPs binding the same transcript and not a direct physical interaction. To test this possibility, the co-immunoprecipitation experiments were performed in animals that express the ADR-1 dsRBD1 mutant that lacks the ability to bind RNA *in vivo* [22, 39]. Similar to the wild-type animals, VIG-1 was detected in the ADR-1 IPs from ADR-1 dsRBD1 mutant animals (Fig. 6A, third lane) and those same animals treated with RNAi to *gsk-3* (Fig. 6A, fourth lane). These results indicate that ADR-1 and VIG-1 physically interact to form a complex, independent of RNA, and GSK-3 does not impact this interaction.

These data raise the possibility that GSK-3 phosphorylates VIG-1 and/or ADR-1, which prevents the RBPs from binding to the regulon. To test this, phosphorylation of ADR-1 and VIG-1 were examined using a general phosphoserine/threonine antibody. To avoid detecting all phosphorylated proteins in *C. elegans*, prior to immunoblotting, the ADR-1–VIG-1 complex was isolated via co-immunoprecipitation as in Fig. 6A. A band corresponding to VIG-1 was detected with the phosphoserine/threonine antibody in the ADR-1 IP from wild-type animals (Fig. 6B). Further, this band was not detected in ADR-1 immunoprecipitations performed from animals treated with RNAi to *gsk-3* or *vig-1* (Fig. 6B). Together, these data suggest that VIG-1 is phosphorylated by GSK-3.

To test if GSK-3 phosphorylation of VIG-1 would impact binding of the ADR-1–VIG-1 complex to the regulon, RBP binding to the regulon was monitored in animals treated with Laduviglusib, a drug that has previously been shown to inhibit GSK-3 kinase activity in *C. elegans* [29]. To confirm that GSK-3 kinase activity was inhibited, transcriptional activity of SKN-1, a protein known to be inhibited by GSK-3 phosphorylation was measured. As expected, in the presence of Laduviglusib, there was a significant increase in expression of the SKN-1 activated gene *gst-4* [51] (Supplementary Fig. S9). Going forward, the impacts of the kinase activity of GSK-3 on ADR-1–VIG-1 binding to the regulon were examined using *pqm-1* and *nhr-193* pull downs from lysates of wild-type animals in the presence and absence of Laduviglusib. As expected, ADR-1 and VIG-1 were not detected in the *pqm-1* and *nhr-193* pulldown samples from wild-type animals in the absence of the inhibitor (Fig. 6C). However, both ADR-1 and VIG-1 were detected in the pulldowns when the animals were treated with Laduviglusib (Fig. 6C). These results demonstrate that phosphorylation of VIG-1 by GSK-3 inhibits the VIG-1–ADR-1 complex from binding to the neural regulon transcripts.

Since loss of GSK-3 kinase activity resulted in VIG-1–ADR-1 complex binding to the regulon genes, and VIG-1–ADR-1 binding leads to downregulation of the regulon, it is expected that expression of the neural regulon is regulated by the kinase activity of GSK-3. To examine this possibility, we sought to measure neural mRNA expression in animals in the presence and absence of Laduviglusib. For these experiments, we used an approach where a 3x FLAG tag is present on poly(A)-binding protein (PAB-1) expressed in animals under the control of a neural promoter, and PAB-1 RIP qPCR can be performed to query transcript status [27, 52]. To validate this approach, neural RIP qPCRs were performed for a muscle-specific gene, *myo-3*, a germline-specific gene, *glh-1* , and a neural-specific gene *unc-64*. While there was more than a 5-fold enrichment for *unc-64* in the neural PAB-1 IP samples (Supplementary Fig. S10), there was no enrichment for *myo-3* or *glh-1* in the PAB-1 IP samples (Supplementary Fig. S10) compared to the input levels of each gene.

With this approach in hand, neural PAB-1 RIPs were performed in animals in the presence and absence of Laduviglusib. Immunoblotting revealed that PAB-1 was immunoprecipitated to a similar extent in these conditions (Fig. 6D). Compared to the input levels, there was a significant enrichment for the regulon genes in the PAB-1 IPs in the absence of Laduviglusib (Fig. 6D). In contrast, in the presence of Laduviglusib, there was a significant reduction in enrichment of the regulon in the PAB-1 IPs (Fig. 6D). These results suggest that in the absence of GSK-3 kinase activity, the regulon genes are downregulated in neural cells. It is important to note, that there was no significant change in *unc-64* enrichment in the presence of Laduviglusib (Supplementary Fig. S10). Together, these results reveal that loss of phosphorylation of VIG-1 by GSK-3 promotes VIG-1–ADR-1 binding specifically to the regulon transcripts, which in turn leads to the downregulation of these mRNAs in neural cells.

## Discussion

In these studies, we uncovered a tissue-specific, post-transcriptional mechanism that regulates expression of a cohort of transcripts that function in lipid metabolism. An unbiased genetic screen and subsequent loss of function analysis revealed that the presence of the kinase, GSK-3, in neural cells, prevents ADR-1 binding specifically to these transcripts in wild-type animals. Mass spectrometry analysis and RNA pulldown studies identified VIG-1 as a second RNA-binding protein bound to the same transcripts in a GSK-3-dependent manner. Additional experiments demonstrated that there is a physical interaction between VIG-1 and ADR-1, and loss of GSK-3 does not impact that interaction. However, VIG-1 was found to be phosphorylated by GSK-3, and the kinase activity of GSK-3 was shown to inhibit ADR-1–VIG-1 binding to the neural regulon transcripts. Thus, together our data reveal a regulatory axis that requires the dsRNA binding protein, ADR-1, the kinase GSK-3 and a second RNA binding protein, VIG-1 to promote the proper expression of lipid metabolism transcripts in the nervous system.

### Phosphorylation of VIG-1 by GSK-3

Our results indicate that VIG-1 is phosphorylated by GSK-3. Further, our data also indicate that in the absence of GSK-3 kinase activity, VIG-1 binds to the regulon. While mechanistic studies of phosphorylation on VIG-1 function have not been performed to date in *C. elegans*, serine/threonine phosphorylation sites on VIG-1 have been identified in large-scale phosphoproteomic studies [53]. In addition, prior studies have shown serine/threonine phosphorylation events on VIG-1 homologs [54] with evidence of inhibition of some cellular functions by phosphorylation [55]. The most common motif for phosphorylation by GSK-3 is S/T-X-X-X-S/T (P), such that GSK-3 phosphorylation occurs on a serine/threonine three residues upstream of a pre-phosphorylated (or “primed”) serine/threonine [56]. While this is the most common GSK-3 phosphorylation motif, there are some studies that show that there could be up to four residues between these phosphorylation sites [30]. On searching for these possible GSK-3 motifs within VIG-1, we found three potential GSK-3 phosphorylation sites, two of which are within the RNA-binding domain of VIG-1 (Supplementary Fig. S11). Future experiments should be aimed at mutating these residues and testing the impacts of these phosphorylation mutants on VIG-1 binding to the regulon. In addition, as GSK-3 acts on primed substrates [56], it is possible that additional kinases function to regulate VIG-1 RNA-binding activity and the post-transcriptional neural regulon.

How ADR-2 regulates the activity of GSK-3, and potentially other kinases, to control VIG-1–ADR-1 RNA binding is an open question. Our unbiased genetic screen revealed that a loss of function mutation in *dsh-2* inhibited ADR-1 binding to the neural regulon in *adr-2 (-)* animals. Since DSH-2 inhibits GSK-3 and our data indicates that inhibition of GSK-3 kinase activity alters VIG-1–ADR-1 RNA binding to the neural regulon, it is likely that *adr-2 (-)* neural cells have decreased GSK-3 activity compared to wild-type neural cells. Further, as GSK-3 is downstream of the WNT receptor and is inhibited by activation of the receptor, it is possible that *adr-2 (-)* neural cells have increased activation of the WNT receptor. To begin to assess this possibility, we compared the neural transcriptome of wild-type and *adr-2 (-)* animals published previously [22]. This revealed significantly increased expression of a single WNT receptor ligand, *cwn-1*, in *adr-2 (-)* neural cells. As *cwn-1* is a WNT receptor activating ligand [57], it is possible that increased expression of this ligand leads to increased activation of the WNT receptor, leading to decreased GSK-3 activity in *adr-2 (-)* neural cells. Since we did not observe ADR-2 binding to *cwn-1* in the nervous system (Supplementary Fig. S12), we still do not understand the molecular mechanism by which ADR-2 regulates *cwn-1* levels. Future studies should be aimed at assessing the molecular mechanism employed by ADR-2 to regulate CWN-1 and other potential WNT activating ligands as well as other signaling pathways linked to GSK-3 activation.

### Connections between lipid metabolism, longevity, and stress resistance

In our studies, we observed that VIG-1 and ADR-1 function together to decrease expression of neural transcripts involved in lipid metabolism. However, the physiological requirement for this post-transcriptional regulation remains unclear. While most of these genes are involved in lipid synthesis, some contribute to lipid breakdown, and yet others have dual roles in both synthesis and breakdown of lipids. Hence, it is possible that the effect of ADR-1 is on the overall balance of lipids as opposed to the specific lipid composition. Several *C. elegans* studies have demonstrated that lipid metabolism is key to adapting to nutrient deficiency [41, 58, 59]. As our studies are performed in larval animals hatched in the absence of food, it is possible the decreased expression of the lipid metabolism genes is important to combat the lack of nutrients. As our prior work demonstrated that ADR-1 binding to *pqm-1* in neural cells is regulated by nutrients [22], and our current findings indicate that the presence of GSK-3 and VIG-1 influence ADR-1 binding; together, this would suggest that the presence of nutrients may impact GSK-3 activity to alter neural expression of the lipid metabolism genes.

While our prior work established that decreased expression of *pqm-1* specifically in neural cells impacted survival to hypoxia (low oxygen) [22], the impact of decreased neural expression of other transcripts of this regulon on organismal physiology are largely unknown. Studies in whole animals have revealed that decreased expression of several of these transcripts (*hyl-1*, *lipl-5, maoc-1*, and *fat-3*) enhanced stress resistance [42–46]. Future studies should focus on specifically modulating levels of these factors in neural cells and assessing the impacts on stress resistance.

Enhanced stress resistance is associated with increased longevity [60], and some studies in invertebrate model organisms and mammals have shown that regulation of neural lipid metabolism is important for longevity [61, 62]. Interestingly, ADARs have also been linked to impacts on aging and longevity [63–65], thus raising the question of whether the effects on neural lipid metabolism gene expression are associated with the longevity phenotypes of *adr* mutant animals. Herein our results indicated that neural cells isolated from *adr-2 (-)* animals exhibited decreased expression of lipid metabolism genes due to ADR-1 binding to these transcripts. Our prior work established that *adr-2 (-)* animals have a longer lifespan than wild-type animals; however, additional loss of *adr-1* reduces the lifespan of *adr-2 (-)* animals to wild-type [63]. Future experiments should determine whether the function of ADR-1 in neural cells is sufficient to alter the longevity phenotype of *adr-2 (-)* animals. Furthermore, as studies about the impact of VIG-1 on longevity are lacking in all organisms, future experiments should explore whether VIG-1 affects *C. elegans* lifespan.

### What is the post-transcriptional regulatory mechanism for decreased neural transcripts?

In our studies, we found that binding of the VIG-1–ADR-1 complex to the regulon leads to downregulation of the regulon. However, the molecular mechanism of downregulation of these transcripts is unknown.

Prior work assessing global RNA expression in young adult *C. elegans* has shown that loss of ADR-1 binding results in upregulation of mRNAs [66]. Additionally, studies in mammals have also demonstrated that ADARs can bind specific mRNAs and downregulate expression [67, 68]. However, the molecular mechanisms for how ADAR binding leads to mRNA downregulation are largely unknown.

Similar to *Drosophila* [69], early biochemical studies identified C*. elegans* VIG-1 as a component of the RNA-induced silencing complex (RISC) [70]. More specifically, biochemical [71] and molecular genetic [70] studies have revealed that VIG-1 is a component of the *let-7* microRNA RISC (miRISC) and is important for silencing of *let-7* target reporter. However, the specific molecular function of VIG-1 in miRNA-mediated gene silencing is unclear. Despite this gap in knowledge, we reasoned that expression of the neural regulon may be regulated by miRISC. However, miRISC composition is complex, and studies have shown that understanding the tissue-specific composition of miRISC is pivotal in determining the fate of target mRNAs [72].

One miRISC factor present in neural cells [73] and in functional complexes with VIG-1 [32] is the RNA helicase, CGH-1. Interestingly, in one replicate of our mass spectrometry analysis, CGH-1 was identified in *pqm-1* pulldowns from animals treated with *gsk-3* RNAi in neural cells but not in animals treated with control RNAi (Supplemental Table S3, tab 4). To determine whether CGH-1 exhibited a similar binding pattern as VIG-1–ADR-1, *pqm-1* and *nhr-193* pulldowns from wild-type animals in the presence and absence of Laduviglusib were subjected to immunoblotting with a CGH-1 antibody. CGH-1 was not observed in the pulldown samples in the absence of Laduviglusib (Supplementary Fig. S13). However, CGH-1 was present in the pulldown samples in the presence of the drug (Supplementary Fig. S13A). These results indicate that, similar to VIG-1–ADR-1, CGH-1 is present on the regulon transcripts in a GSK-3-kinase dependent manner.

To test whether the regulon genes are regulated by CGH-1, we tested whether knocking down *cgh-1* impacted *dod-24* expression in a GSK-3-dependent manner. As shown previously, compared to control, animals treated with *gsk-3* RNAi in neural cells exhibited decreased *dod-24* expression (Fig. 4A) and animals with RNAi against *gsk-3* and *vig-1* in neural cells exhibited a significant increase in *dod-24* expression (Fig. 5B and Supplementary Fig. S13B). Interestingly, a similar rescue in *dod-24* expression was observed upon knocking down *cgh-1* in the absence of *gsk-3* (Supplementary Fig. S13B). Furthermore, additional treatment with *cgh-1* RNAi did not significantly alter *dod-24* expression in animals treated with *gsk-3* and *vig-1* RNAi (Supplementary Fig. S13B). These results suggest that expression of the neural regulon may be mediated by miRISC. Furthermore, as somatic RISC promotes mRNA deadenylation and decay [74], a potential molecular mechanism for the downregulation of the neural regulon is via miRISC-dependent mRNA cleavage. Future experiments should be aimed at determining the miRNAs, Argonautes, and other protein components of miRISC in the *C. elegans* nervous system and monitoring the impacts of these additional factors on expression of the neural regulon.

## Supplementary Material

gkaf785_Supplemental_Files

## Data Availability

Raw and processed high-throughput sequencing data generated in this study for RIP sequencing and neural RNA sequencing experiments have been submitted to the NCBI Gene Expression Omnibus under accession numbers GSE286246 and GSE286247 respectively (https://doi.org/10.5281/zenodo.16378230). High-throughput sequencing data generated for the EMS mutagenesis experiment have been submitted to the NCBI Sequence Read Archive under accession number PRJNA1208471. Raw and processed mass spectrometry data have been uploaded to MassIVE with accession MSV000097188 and cross-referenced to ProteomeXchange PXD061064. The in-depth code that was followed is available on GitHub: https://github.com/ananya716/GSF3874-EMS-pilot/blob/main/Restarting%20from%20alignment (https://doi.org/10.5281/zenodo.16364942).
