# Supplementary material for: The kinase GSK-3 alters the RNA-binding protein landscape of lipid metabolism transcripts leading to altered expression in the C. elegans nervous system
Source: Nucleic Acids Res. 2025 Aug 19;53(15):gkaf785. doi: 10.1093/nar/gkaf785 (PMC12362252; doi:10.1093/nar/gkaf785)
Supplement: gkaf785_Supplemental_Files [file gkaf785_supplemental_files.zip › Supplementary Information.pdf]

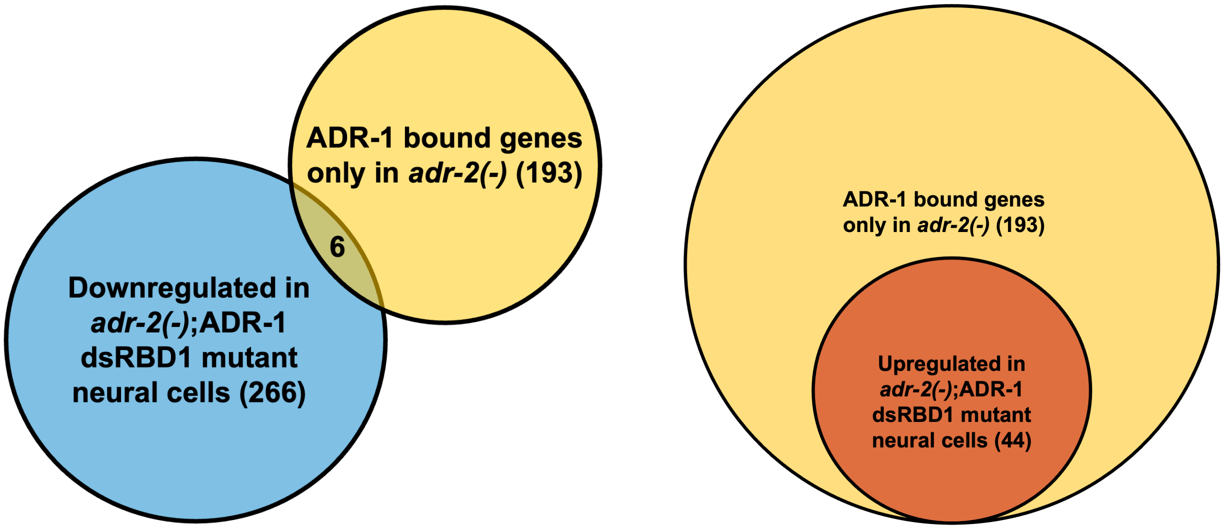

Supplementary Figure S1: Overlap between ADR-1 bound genes only in the absence of *adr-2* and all the mis expressed genes in *adr-2(-)*;ADR-1 dsRBD1 mutant neural cells compared to *adr-2(-)* neural cells.

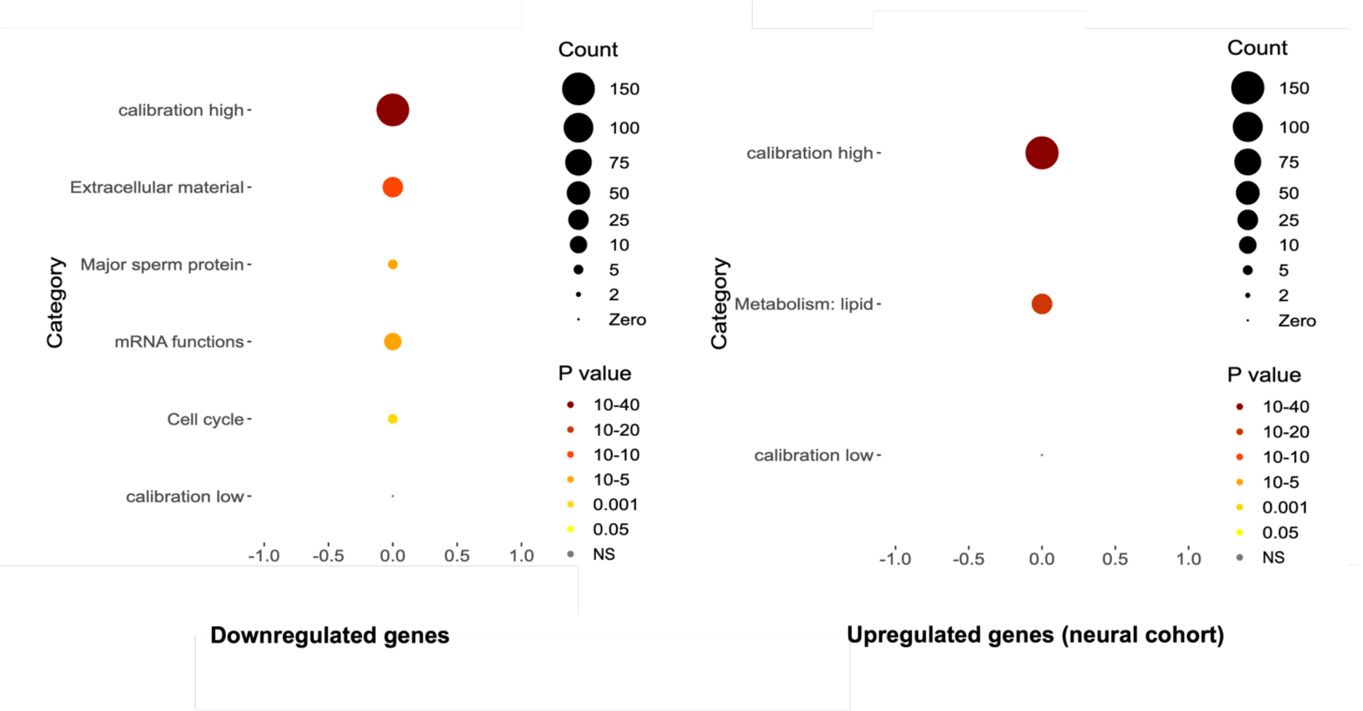

Supplementary Figure S2: WormCat gene set enrichment analysis for all the differentially regulated genes in *adr-2(-)*;ADR-1 dsRBD1 mutant neural cells compared to *adr-2(-)* neural cells. For the total number of genes input into the software (regulated gene set), the *p* value is calculated using the Fisher's exact test. "Count" represents the number of genes within the specific category (only one category obtained). The size of the circles indicates the number of genes in that specific category and the color of the circles indicates the *p* value (key in figure).

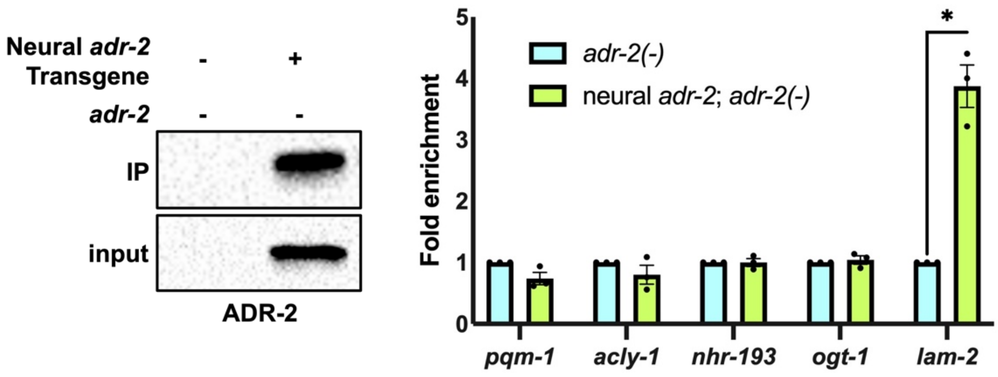

Supplementary Figure S3: ADR-2 does not bind to the regulon in the nervous system. Left- Lysates and immunoprecipitates from the indicated strains were subjected to immunoblotting with a FLAG antibody. Blot is a representative image from three independent biological replicates. Right- Plotted bar graphs represent the fold enrichment of cDNA of indicated genes in the neural ADR-2 IP samples compared to the input samples from animals indicated. The IP/input values were normalized to the calculated value for the negative control *adr-2*(-) animals. The mean of 3 biological replicates was plotted. Statistical significance was calculated by multiple unpaired *t* tests followed by Holm–Šídák multiple comparisons correction. *$p < 0.01$.

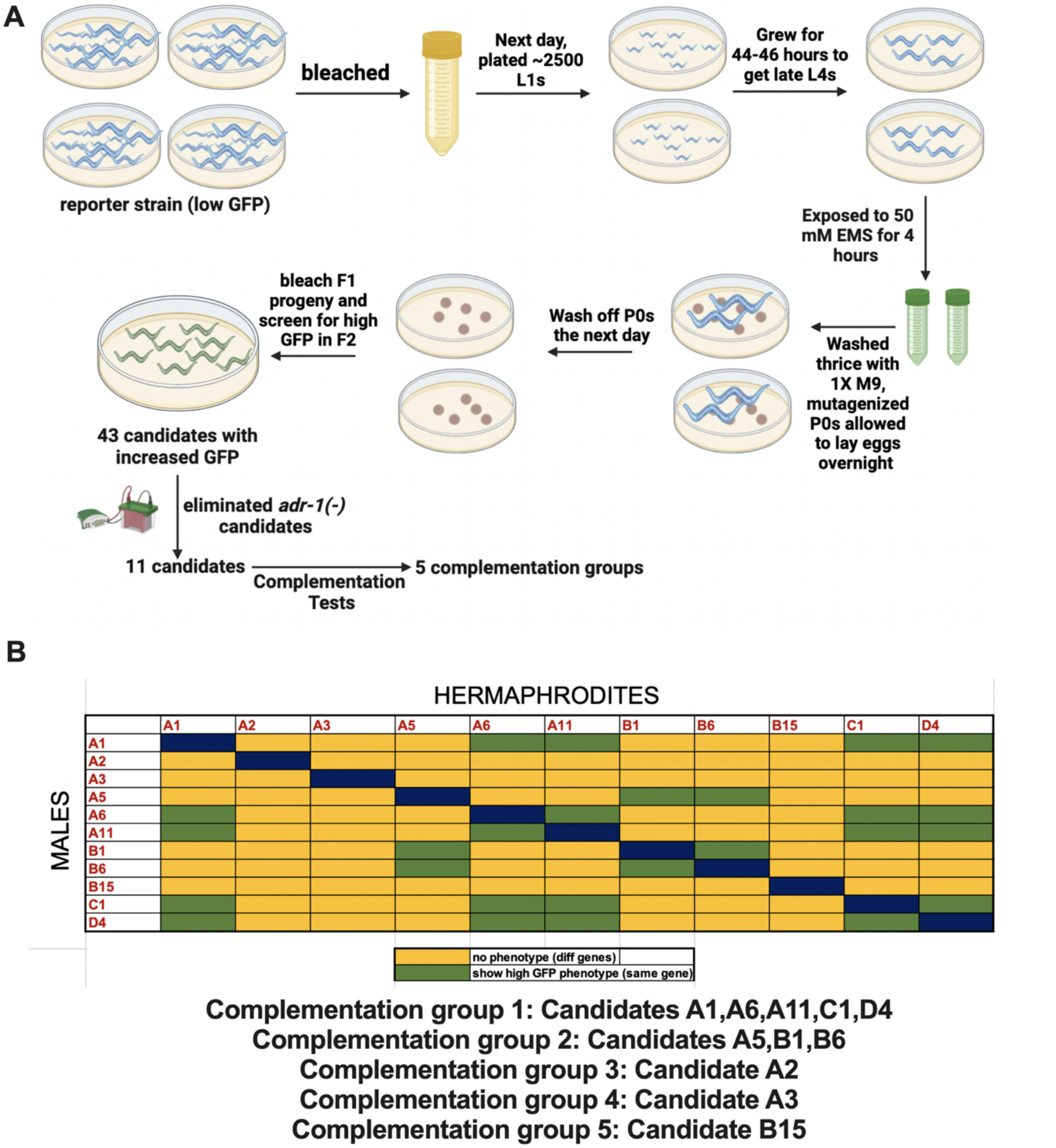

Supplementary Figure S4: Schematic of EMS mutagenesis screening. Strain used for mutagenesis is a GFP transcriptional reporter of the PQM-1 activated gene *dod-24.* Strain has low GFP due to decreased *dod-24* expression. Animals were screened for high GFP levels and ADR-1 expression in candidates was monitored using Western blot after which *adr-1(-)* candidates were eliminated. Completion of mutant complementation tests with candidates resulted in 5 unique complementation groups.

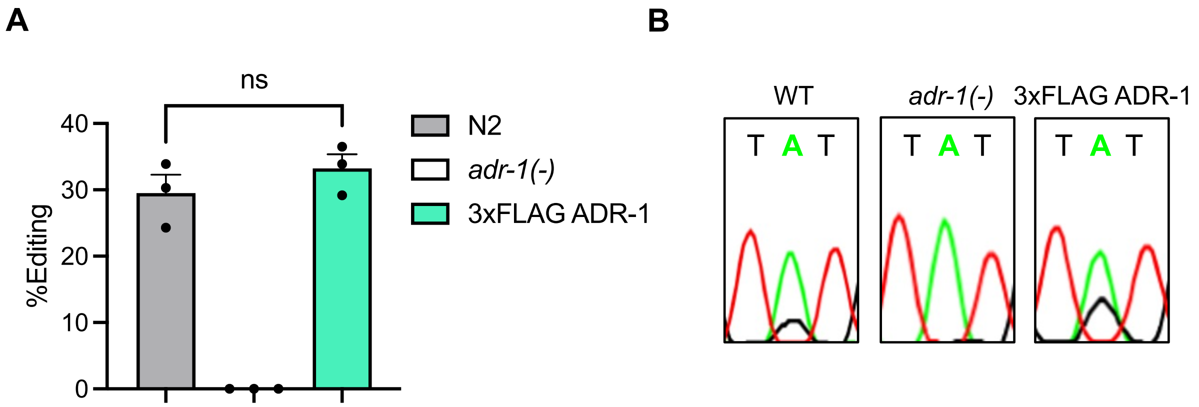

Supplementary Figure S5: Insertion of 3X FLAG tag to ADR-1 does not impact ADR-1 function of regulating editing by ADR-2. (A) An editing assay was performed to monitor A-to-I editing levels of a known edited site in *lam-2*. The bar graphs indicate the quantified editing levels at the site measured from the Sanger sequencing traces from three biological replicates. Error bars represent the standard error of mean (SEM). Statistical significance was calculated by performing unpaired t-tests (ns = not significant (p>0.05)). (B) Sanger sequencing traces are indicated on the right (green trace = A, black trace = G, red trace = T).

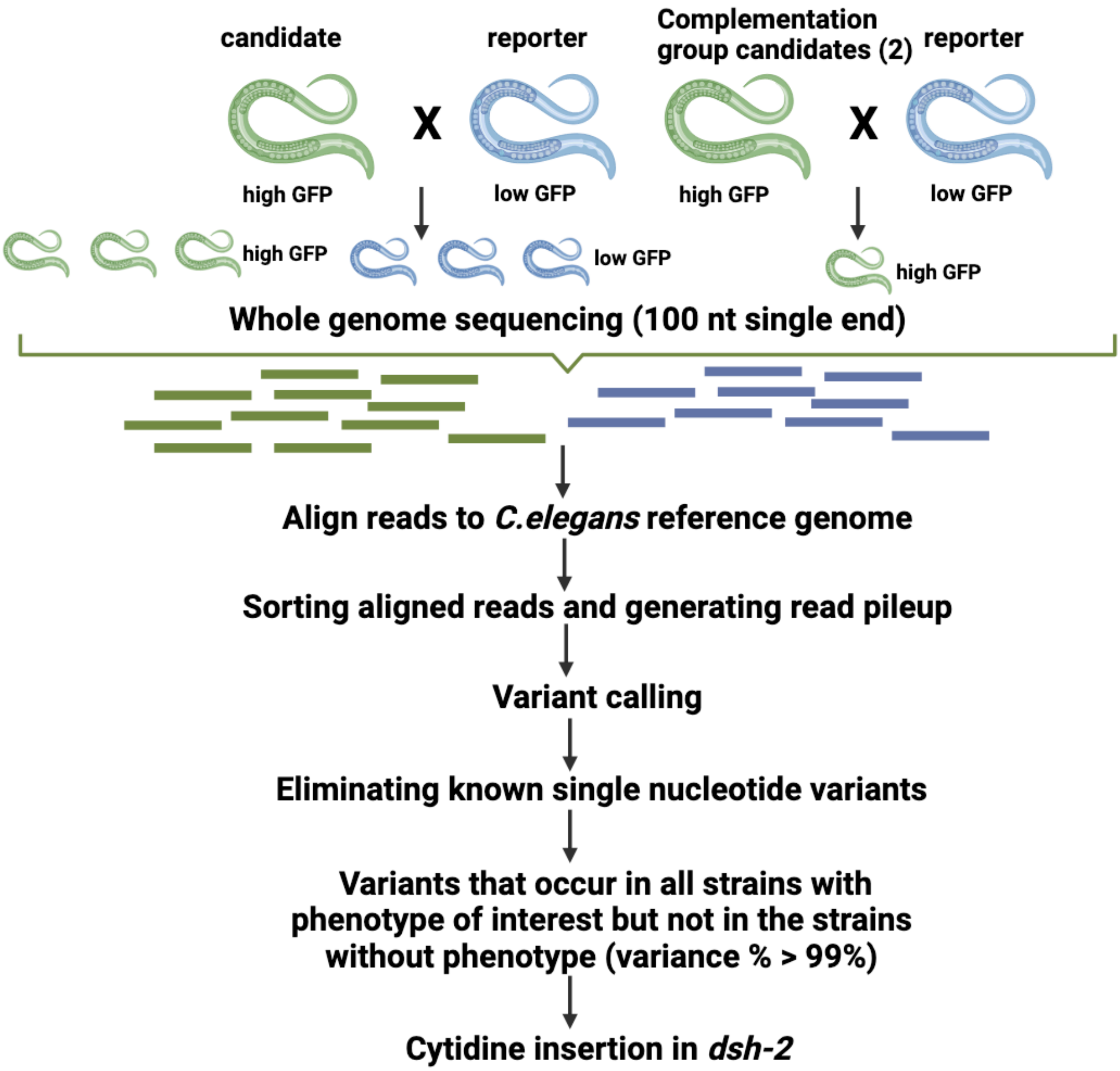

Supplementary Figure S6: Bioinformatics pipeline used to identify causative mutation. In the complementation group with three candidates (A5, B1 and B6), three strains with phenotype of interest and three strains without phenotype of interest were isolated from one candidate (A5). For the other two candidates within the same complementation group (B1 and B6), one strain with the phenotype of interest was isolated, and all these animals were subjected to whole genome sequencing.

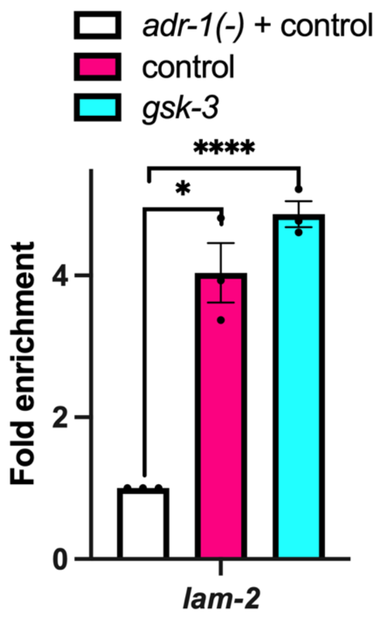

Supplementary Figure S7: ADR-1 binding to *lam-2* is not affected by GSK-3 RNAi in neural cells. Plotted bar graphs represent the fold enrichment of cDNA of indicated gene in the IP samples compared to the input samples from animals treated with indicated RNAi conditions in neural cells. The IP/input values were normalized to the calculated value for the negative control *adr-1*(-) + control RNAi animals. The mean of 3 biological replicates was plotted. Error bars represent SEM. Statistical significance was calculated by multiple unpaired *t* tests followed by Holm–Šídák multiple comparisons correction. *$p < 0.05$, ****$p < 0.0001$.

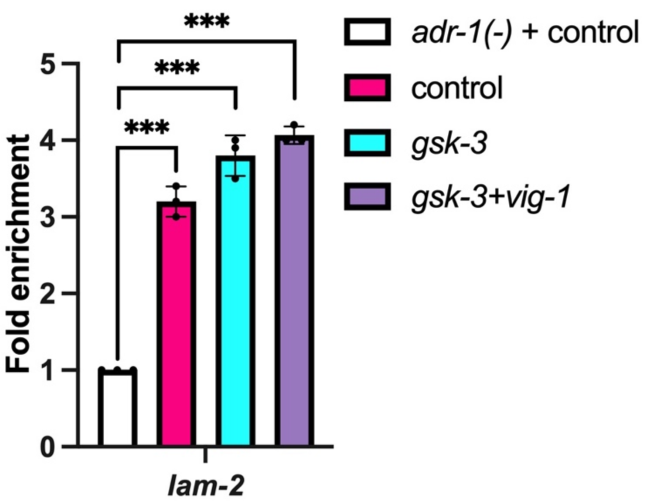

Supplementary Figure S8: ADR-1 binding to *lam-2* is not affected by GSK-3 and VIG-1 RNAi in neural cells. Plotted bar graphs represent the fold enrichment of cDNA of indicated gene in the IP samples compared to the input samples from animals treated with indicated RNAi conditions in neural cells. The IP/input values were obtained and normalized to the calculated value for the negative control *adr-1*(-) + control RNAi animals. The mean of 3 biological replicates was plotted. Error bars represent SEM. Statistical significance was calculated by multiple unpaired *t* tests followed by Holm–Šídák multiple comparisons correction. ***$p < 0.0001$.

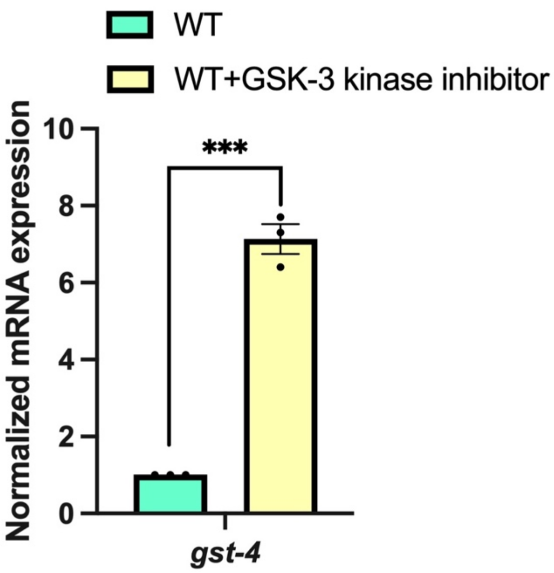

Supplementary Figure S9: Expression of *gst-4* as a measure of SKN-1 transcriptional activity in the presence and absence of GSK-3 kinase inhibition. Gene expression measured by qPCR. Expression of indicated gene was determined relative to expression of the housekeeping gene *gpd-3*. Values were then normalized to wildtype. The bar graphs represent the mean of 3 biological replicates. Statistical significance was calculated using multiple unpaired *t* tests followed by Holm–Šídák multiple comparisons correction. ***$p < 0.0005$.

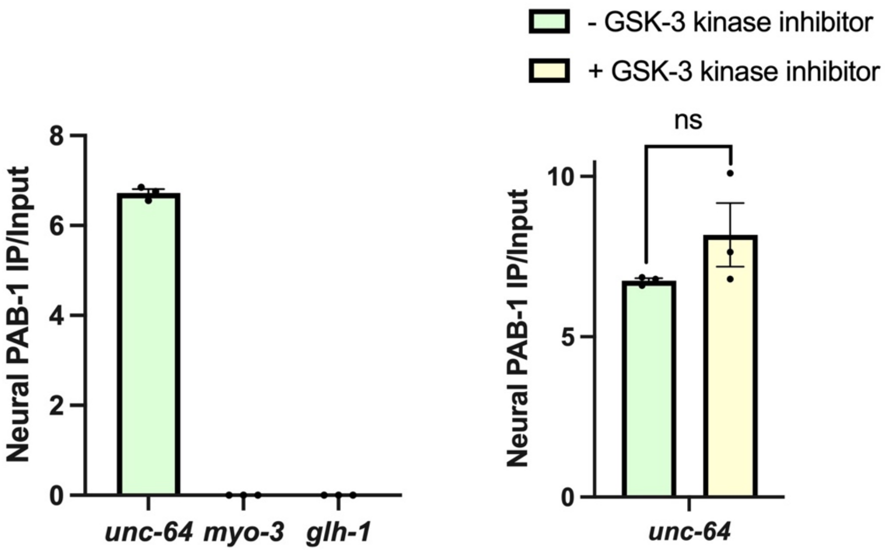

Supplementary Figure S10: Enrichment of neural genes but not germline or muscle genes observed in the neural PAB-1 pulldowns. The graphs represent the neural PAB-1 IP/input values of the cDNA of the indicated genes in the presence and absence of Laduviglusib. The mean of 3 biological replicates was plotted. Error bars represent SEM.

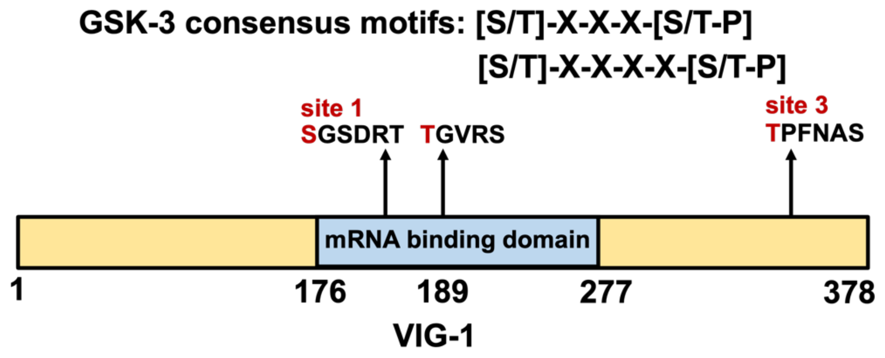

Supplementary Figure S11: Uniprot domain structure of VIG-1 with potential GSK-3 phosphorylation sites indicated.

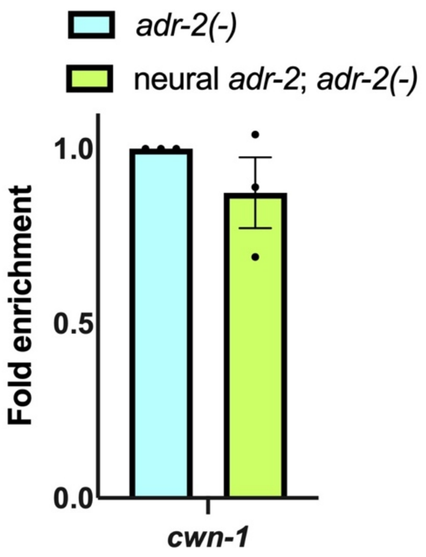

Supplementary Figure S12: ADR-2 does not bind to WNT ligand *cwn-1* in the nervous system. Plotted bar graphs represent the fold enrichment of cDNA of indicated gene in the neural ADR-2 IP samples compared to the input samples from animals indicated. The IP/input values were normalized to the calculated value for the negative control *adr-2*(-) animals. The mean of 3 biological replicates was plotted. Error bars represent SEM.

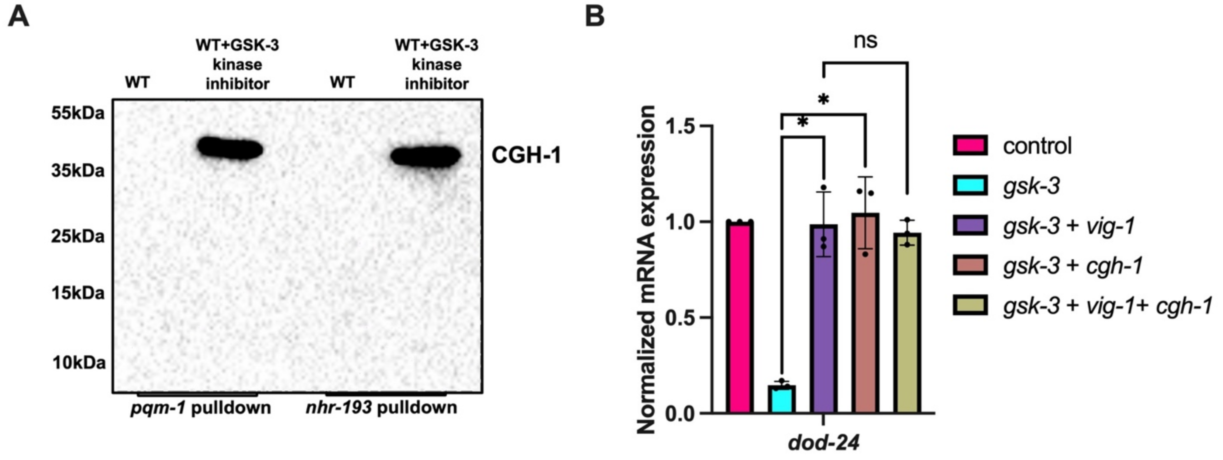

Supplementary Figure S13: CGH-1 associates with the regulon genes and impacts *dod-24* expression in a GSK-3-dependent manner (A) *pqm-1* and *nhr-193* pulldown samples from wildtype animals in the presence and absence of GSK-3 kinase inhibitor Laduviglusib were subjected to immunoblotting with a CGH-1 antibody. Blot is a representative image from three independent biological replicates. (B) Gene expression of L1 animals measured by qPCR after treating animals with RNAi against indicated genes in neural cells. Expression of *dod-24* was determined relative to expression of the housekeeping gene *gpd-3*. Values were normalized to control neuronal RNAi treated animals. The bar graph represents the mean of 3 biological replicates. Statistical significance was calculated using multiple unpaired *t* tests followed by Holm–Šídák multiple comparisons correction. *$p < 0.05$. Error bars represent SEM.
